# Comparative molecular dynamics studies of heterozygous open reading frames of DNA polymerase eta (η) in pathogenic yeast *Candida albicans*

**DOI:** 10.1038/srep41087

**Published:** 2017-01-25

**Authors:** Suresh Satpati, Kodavati Manohar, Narottam Acharya, Anshuman Dixit

**Affiliations:** 1 Department of translational research and technology development, Computational Biology and Bioinformatics laboratory, Institute of Life Sciences, Bhubaneswar, 751023, Odisha, India; 2 Department of Infectious Disease Biology, Laboratory of Genomic instability and Diseases, Institute of Life Sciences, Bhubaneswar, 751023, Odisha, India

## Abstract

Genomic instability in *Candida albicans* is believed to play a crucial role in fungal pathogenesis. DNA polymerases contribute significantly to stability of any genome. Although Candida Genome database predicts presence of *S. cerevisiae* DNA polymerase orthologs; functional and structural characterizations of Candida DNA polymerases are still unexplored. DNA polymerase eta (Polη) is unique as it promotes efficient bypass of cyclobutane pyrimidine dimers. Interestingly, *C. albicans* is heterozygous in carrying two Polη genes and the nucleotide substitutions were found only in the ORFs. As allelic differences often result in functional differences of the encoded proteins, comparative analyses of structural models and molecular dynamic simulations were performed to characterize these orthologs of DNA Polη. Overall structures of both the ORFs remain conserved except subtle differences in the palm and PAD domains. The complementation analysis showed that both the ORFs equally suppressed UV sensitivity of yeast *rad30* deletion strain. Our study has predicted two novel molecular interactions, a highly conserved molecular tetrad of salt bridges and a series of π–π interactions spanning from thumb to PAD. This study suggests these ORFs as the homologues of yeast Polη, and due to its heterogeneity in *C. albicans* they may play a significant role in pathogenicity.

*Candida albicans (C. albicans)* is an opportunistic fungus that usually found as harmless commensals in humans^1^. In immunocompetent hosts, because of the active immune responses against *Candida* antigens, *C. albicans* mostly superficially colonizes without causing any noticeable diseases^2^. However, in severely immunocompromised individuals *C. albicans* causes systemic infection^3^,^4^. The apparent increase of fungal infections is directly correlated to the proportionate increase in the number of immunocompromised patients, and to increasing resistance to available fungal drugs^5^. *C. albicans* is perpetually a diploid and is mostly dimorphic^6^,^7^. The genome of *C. albicans* consists of eight pairs of homologous chromosomes that exhibit a high degree of natural heterozygosity^8^,^9^. Several studies have demonstrated that allelic differences result in structural and functional differences in the encoded proteins^10^,^11^,^12^,^13^. In addition to morphological switching, genomic instability in forms of genomic mutation, gain or loss of chromosomes, and loss of heterozygosity (LOH) have been reported in many clinical isolates; and is often associated with virulence and drug resistance of *Candida*^14^,^15^,^16^.

The function of any genome solely depends upon efficient and accurate replication of the complementary DNA strands by DNA polymerases as DNA replication defects are the major source of genomic instability in the cell^17^,^18^,^19^. Elucidation of the role of various DNA polymerases in *C. albicans* pathogenesis is critical to understand genomic instability associated fungal infections and ways to prevent or control them. Although, Candida Genome database (CGD) annotates presence of yeast DNA polymerase orthologs, none of the DNA polymerases have been fully characterized yet^8^. DNA polymerase eta (Polη) is unique among DNA polymerases in its ability to promote proficient and error-free replication through UV-induced cyclobutane pyrimidine dimers (CPDs)^20^,^21^. Inactivation of Polη in both yeast and humans confers enhanced UV mutagenesis and in humans, causes the cancer-prone syndrome, the variant form of xeroderma pigmentosum^22^,^23^. Co-crystal structure of Polη with CPD containing substrate revealed that Polη has the unique ability to accommodate two Ts of covalently linked TT of CPD in its wide catalytic center, unlike any other DNA polymerase^24^,^25^. The open right handed structure of Polη stabilizes DNA template-primer with its palm, fingers, thumb and polymerase-associated domain (PAD) domains. The palm carries the active site acidic residues that form a catalytic triad to catalyze the nucleotidyl transfer reaction in the presence of two Mg^2+^ ions.

Interestingly, according to CGD *C. albicans* is heterozygous in possessing Polη with two distinct ORFs *viz.* CaO19.866 (hereafter referred as ORF1) & CaO19.8485 (ORF2)^8^. Both the ORFs belong to the chromosome #2 at same locus with a single copy but differ only by three amino acids. The promoter sequences of the Polη alleles are found to be identical and thus, probably the mRNA expression will not differ significantly. As allelic differences often result in structural and functional differences in the encoded proteins, we have taken an *in silico* approach to characterize these orthologs as DNA Polη and kinds of structural differences they might possess to impact their function. Since, there are no reports on *C. albicans* Polη, we developed 3D protein homology models for the two ORFs using *S. cerevisiae* Polη as a template. Moreover, we have carried out a 240 nanosecond (ns) molecular dynamics simulation to compare the dynamic behaviour of these structures. Our understandings of structure-function relationship and an insight into the dynamic behaviour of both the ORFs are discussed.

## Results

### Specie-wise residue conservation

**Figure 1:**
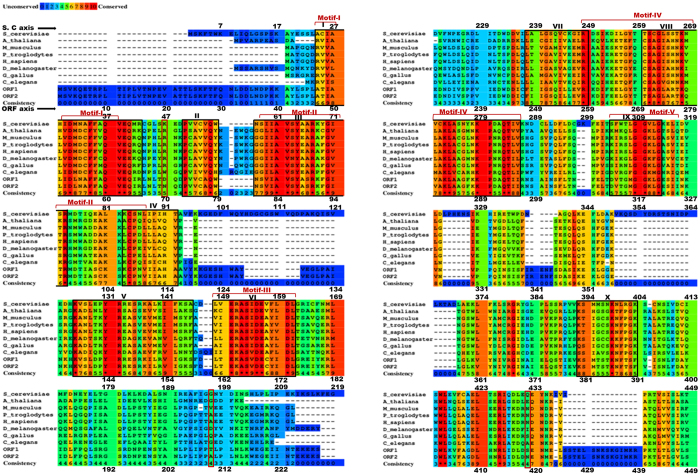
Multiple sequence alignment of Polƞ sequences from different species. The sequence alignment was performed between nine different species namely *Pan troglodytes, Mus musculus, Gallus gallus, Drosophila melanogaster, Arabidopsis thaliana, Caenorhabditis elegans, Homo sapiens, Saccharomyces cerevisiae and Candida albicans* (ORF1 and ORF2) using Praline software. The residues are coloured by the conservation index (consistency scale) where identical residues are shown by ‘*’ mark. The black coloured boxes highlights the conserved patches (patch I–X) and red coloured square braces indicates motifs (I–V) which are also part of the patches. The red coloured boxes highlight the three residues that vary among the two Candida ORFs.

**Figure 2:**
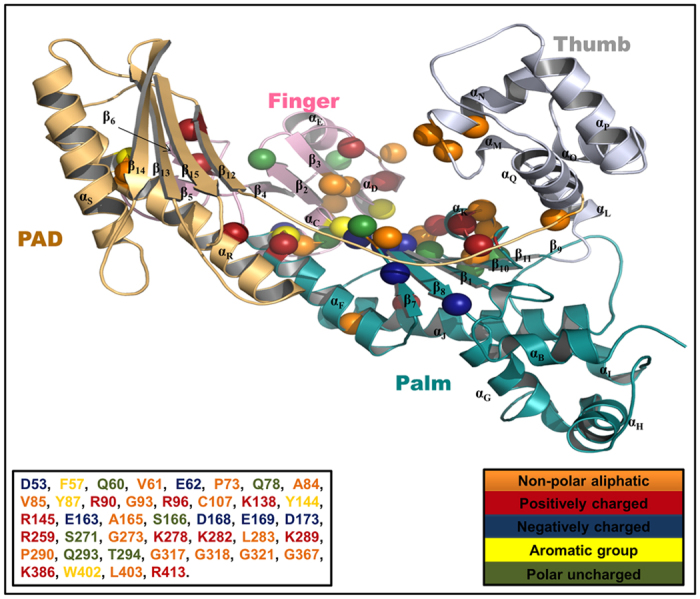
The schematic illustration of identical residues among species. The identical amino acids are represented on the four different domains of the Polη 3D structure. Each domain is coloured differently i.e. Palm–cyan, PAD–golden, Thumb–white and Finger–magenta. The protein secondary structures are labelled as per the *S. cerevisiae* nomenclature. The residues are shown in sphere and coloured according to their chemical properties as shown. The amino acids are numbered as per *Candida* ORF sequence.

The residues which are conserved during the evolution usually have functional relevance. Analysis of such residues can give valuable insights into the functioning of the DNA polymerases. Therefore, to identify conserved residues in Polη, a multiple sequences alignment was performed using Polη sequences from many different diverse species (Fig. 1 and Supplementary Fig. 1(A–D)). We identified a total of 41 identical residues across the species (Supplementary Table S1). It was interesting to note that the finger domain has the highest number of such residues. It was followed by the palm and then the thumb domain. All the identical residues are shown as a sphere on the Polη structure (Fig. 2). Each domain is represented in a different colour; the domain names were assigned according to *S. cerevisiae* Polη nomenclature. Mutational analyses of some of these conserved residues e.g. A84, Y87, R96, D168, E169 in yeast and human have already shown their importance in catalysis and fidelity of Polη^21^,^26^,^27^,^28^,^29^.

Further analyses of the conserved residues led us to the identification of conserved patches in Polη sequences (black coloured boxes in Fig. 1). The patches were identified on the basis of the following criteria (i) residue conservation index greater than five and, (ii) a stretch of at least seven amino acids. We identified ten such patches in the Polη family; these are I (47–65 (β_1_-α_C_)), II (73–79 (β_2_)), III (80–96 (β_3_-α_D_)), IV (106–114 (β_4_)), V (138–158 (β_6_-α_F_)), VI (161–174 (β_7_-β_8_)), VII (248–259(α_J_), VIII (270–296 (β_10_-α_K_-β_11_)), IX (312–321 (α_N_)), X (380–391(β_12_)) (Supplementary Fig. 1E). Out of these, five patches correspond to earlier identified conserved motifs I-V (highlighted in Fig. 1). The motif-I resides in between β_1_ of palm and α_C_ of finger domains, motif-II is present at the β-sheet (β_3_) and α-helix (α_D_) on the tip of the finger domain. The β-sheet (β_7_ & β_8_) of palm domain harbours the motif-III, while motif-IV is present at the lower α-helix-β-sheet- α-helix (α_J_-β_10_- α_K_) of palm and motif-V is present at the α-helix (α_N_) of thumb domain facing DNA-binding cleft^21^,^24^. The strategic positioning of these motifs in different domains highlights their importance for the polymerase activity. In these motifs, the acidic residues i.e. D168 (β_7_), E169 (β_8_) present at β hairpin and D53 (β_1_) at motif-III bind to Mg^2+^ ions while K289 (α_K_) present at α-helix of motif-IV are critical for nucleotidyl trasferase reaction. The aromatic amino acids i.e. F58 (α_C_) present at motif-I and Y87 (α_D_) & R90 (α_D_) present at motif-II in the finger domain align the incoming dNTP to the templating DNA base while E62 (α_C_) helps in maintaining the structural integrity of finger domain. It is interesting to note that these five motifs contain 31 conserved residues out of total 41. Additionally, a motif has either positively or negatively charged conserved residues but not both. For instance motif-I and III have negatively charged conserved residues only, similarly motif-II and IV have positively charged residues only. The motif-V does not have any charged residue that is conserved (Supplementary Table S1). Extraordinary conservation of these residues across the species suggested that the overall structure and mechanism of DNA synthesis by Polη remains the same during the course of evolution^30^,^31^. The thumb and PAD domains of Polη vary widely among the species. It is intriguing that the three amino acids that differ between ORF1 and ORF2 (red coloured box in Fig. 1) do not belong to any of the conserved patches/motifs.

### Evaluation of structural stability

To check the structural stability of each *C. albicans* ORF throughout the dynamics, we have performed the Root Mean Square Deviation (RMSD) calculations for the protein backbone atoms. The RMSD curves for the ORFs are shown in Supplementary Fig. 2 (ORF1-blue, ORF2-red) indicate that both the systems were evolving until ~20 ns and thereafter the structures remained stable within an average RMSD of ~2 Å. It can be fairly assumed at this point that both systems are stable and there is no drastic conformational change. Therefore, all further calculations for both of the systems were done on the last 60 ns MD trajectory. A comparison of the RMSD values for the two ORFs indicated that ORF1 is more stable conformationally as compared to ORF2. This stabilization comes from the fact that there is a difference of three residues among the two ORFs. Further, to assess the stability of DNA; an RMSD analysis for DNA backbone atoms was performed. As indicated by the RMSD, the DNA was found to be stable (RMSD < 2.0 Å) in both of the simulations (Supplementary Fig. 3).

### Flexibility analysis

**Figure 3:**
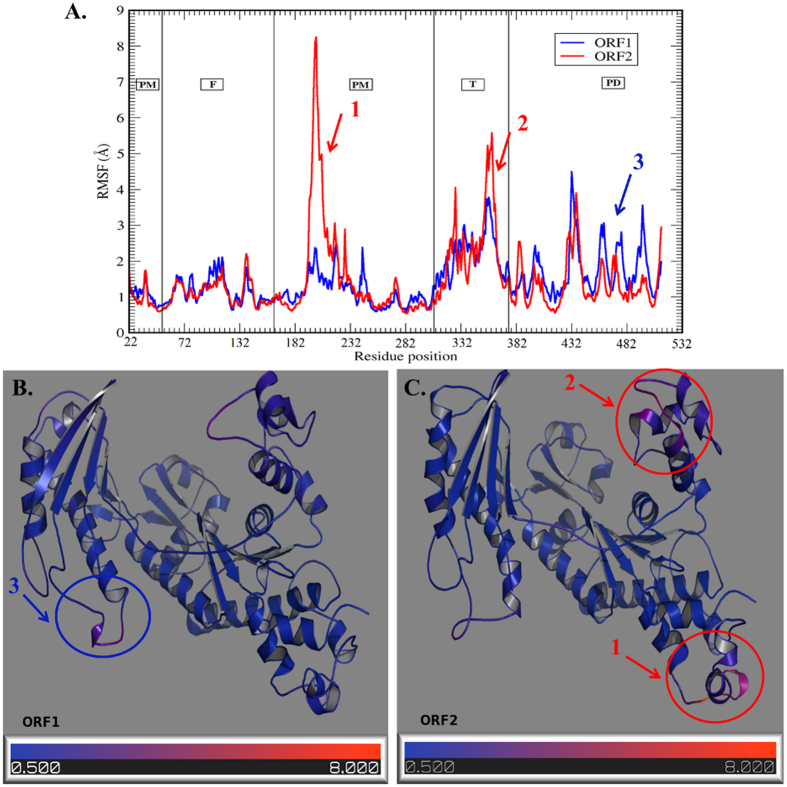
The root mean square fluctuation (RMSF) plot based on (C_α_) profiles. (**A**) The RMSF plot. The residue number and RMSF(Å) is shown on ‘X’ and ‘Y’ axis respectively. The area of each domain is highlighted in a rectangular box (PM–Palm, F–Finger, T–Thumb and PD–PAD domain. (**B**,**C**) The RMSF values plotted on ORF structures. The colour key represents the range of protein flexibility values from lowest to highest flexibility i.e. Blue (0.5)–Red (8.0). The three major differences in palm, thumb and PAD domain are highlighted on ORF structures.

To gain an insight in the fluctuations of the amino acid residues, RMSF for the Cα atoms of each ORF was calculated. The RMSF curves are shown in Fig. 3A (ORF1-blue, ORF2-red). To better visualize regions of high and low flexibility, the RMSF was projected on the 3D modeled structures (Fig. 3B,C). The residues are coloured from blue to red representing low to high RMSF values respectively. The RMSF data showed that the finger domain, as compared to others, is least flexible in both of the ORFs. It is also interesting that the finger domain has the highest number of evolutionarily conserved residues that also makes it critical for the activity of the Polη. The substitution of Asp70 (ORF1) to Asn (ORF2) in finger domain did not result to any significant difference in flexibility of this domain. The two ORFs show varying structural flexibility in other three domains. Comparatively, the palm and thumb domains showed higher flexibility in ORF2 while PAD domain showed higher flexibility in ORF1. The maximum variation in flexibility was observed at the lower palm domain (residue 187–197), although the amino acid sequence of both the ORFs at this stretch is identical. However, as there is a single residue substitution in ORF2 (Thr210 to Ala) which is nearby and may have a role in the observed variation in the flexibility. The RMSF plot also highlighted greater flexibility in ORF2 at thumb domain for two stretches of residues (317–322 & 342–352). The substitution of Ser419 (ORF1) to Asn (ORF2) in the PAD domain might lead to better hydrogen bond networking with nearby residues that is reflected in a slight decrease in flexibility near residues 442–492 in ORF2.

### Molecular surface area analysis

The solvent accessible surface area (SASA) of a molecule is the area of the molecular surface that is exposed to the solvent. The free energy of a protein is roughly proportional to the SASA. Therefore, a change in SASA values of a protein may indicate changes in the protein fold. In the current study, the solvent-exposed molecular surface area of the ORF’s structures was calculated for the last 60 ns of the trajectory using a Tcl script in the VMD. The SASA value of individual residues for both the ORFs is shown in Supplementary Fig. 4. The blue and red lines show SASA for ORF1 and ORF2 respectively. The difference in SASA for each residue between the two ORFs is plotted in green. Interestingly, none of the residue showed greater than 100 Å^2^ change in average SASA values. It indicates that both the ORFs have more or less similar SASA values. The overall SASA difference observed in the palm, finger, PAD and thumb domain was ~119 Å^2^, 41 Å^2^, 256 Å^2^ and 556 Å^2^ respectively. Similar to RMSF, the finger showed the least variation in SASA among the domains. The greatest variation among the two ORFs was found in the thumb domain.

### Assessment of protein fold

The radius of gyration (Rg) was used to estimate the overall change in the shape and total compactness of the ORF structures (Supplementary Fig. 5). The Rg for ORF1 and ORF2 was found to be 27.6 Å and 26.8 Å respectively. This emphasizes that protein conformation was stable for both the ORFs throughout the simulation. Further to check the compactness of the two ORFs, the maximum interatomic distance was calculated. To achieve this, we have measured the largest distance between any two atoms in every frame of the trajectory for each ORF. The maximum interatomic distance was found to be 128 Å and 134 Å for ORF1 and ORF2 respectively. This indicated that the overall structure and the spatial arrangement of the residues remained constant during the dynamics run except for minor fluctuations.

### Protein secondary structure analysis

**Figure 4:**
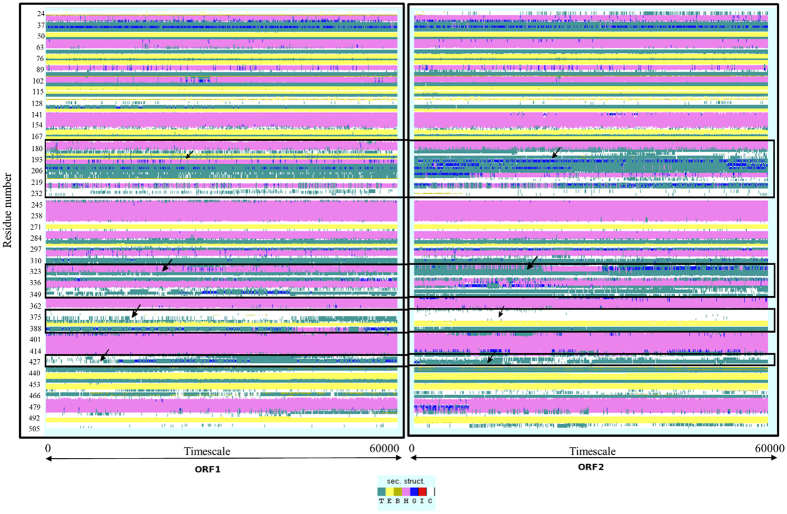
The timeline analysis showing changes in secondary structure. The ‘X’ axis represents time period while ‘Y’ axis represents changes in secondary structure. The highlighted boxes indicate the domains while arrows indicate significant variations in secondary structure. In the secondary structure colour key, the symbol ‘T’ represents hydrogen bonded turn, ‘E’ represents extended β-sheet in parallel or anti-parallel, ‘B’ represents single pair β-bridge, the helices ‘H’, ‘G’ and ‘I’ represents 4, 3, 5-turn helix and ‘C’ represents the coil.

In the timeline analysis, it is easy to identify critical changes in protein structure during dynamics run. It provides greater insight into the changes in the secondary structure. Each secondary structure type is shown by different colour and a change in secondary structure type is easily distinguishable. The secondary structure information for each frame was calculated and a two-dimensional plot was generated for last 60 ns trajectory (Fig. 4). The colour key green-pink-white denotes β sheet-α helix-coil transition over the entire trajectory. It gives a visual depiction of the stable and non-stable secondary structural patterns across the trajectories. A comparison of secondary structural changes during simulation between ORF1 and ORF2 revealed that a stretch of residues (192–219), at the lower palm domain, showed a major transition in the form of α-helix (ORF1) changing to a 3-turn helix in ORF2 (pink to blue transition). Similar transition was observed for residues (323–349) at the thumb domain (alpha helix in ORF1 to 3-turn helix in ORF2) and a local structural change (turn in ORF1 to coil in ORF2) between residues 375–386 at the PAD domain (green to white transition, Supplementary Fig. 6) It is important to note that these structural differences in secondary structure coincide with the residue positions where major changes in the flexibility pattern were observed in RMSF flexibility analysis.

### PCA and clustering analysis

**Figure 5:**
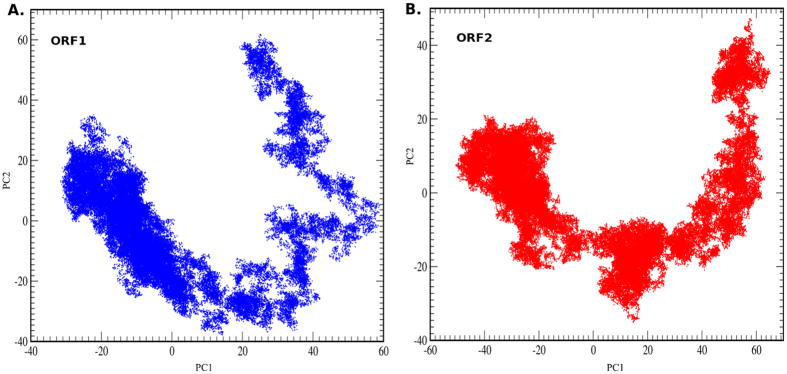
The principal component analysis. The plot between first and second principal component is represented. The plot for ORF1 and ORF2 are shown in blue and red respectively. The abscissa (‘X’) corresponds to PC1 and ordinate (‘Y’) corresponds to PC2. Each system showed nearly five different cluster points.

To get a better insight in the conformational preferences of the two ORFs, a principal component analysis (PCA) was performed on the MD trajectories. Figure 5 illustrates the overall conformational transition occurs throughout the entire dynamics. The PCA uses dynamical state of protein to generate the most dominant native conformational state(s) that cover the entire motion. The most prominent configurational space taken by ORF structures in the MD simulation was projected on a time-dependent scale. The clusters were generated by α-carbon atoms using the first two dominant principal components (Supplementary Fig. 7A). It can be seen (Fig. 5) that a total of five clusters were observed for each ORF during the total dynamics. The pattern of motion depicts that a large portion of structures accumulates as a single cluster for ORF1 and a similar motion is observed for ORF2 but with a comparatively smaller cluster size. To identify a core representative structure from each cluster, the snapshots taken at each 2 ps interval from each trajectory were superimposed and a centroid for each cluster was generated. Additionally, a structural comparison was made between the centroid of the largest cluster with the template (PDB ID: 2WTF) to better interpret the clustering data. (Supplementary Fig. 7B) The comparison between the 3D structure of the representative centroids (ORF1 and ORF2) and template has shown that the greater differences occur at the PAD domain where the β sheets are comparatively small in the centroids (ORF1 and ORF2) than the template. Minor differences were observed at the thumb and palm domain, while finger domain remained unchanged. The helix (α_N_) is shortened in the two ORFs as compared to the template. To visualize the classified clusters, the corresponding RMSD values for each frame were plotted across the two ORFs (Supplementary Fig. 8). Further, the generated clusters were superimposed and checked for relative motions of different regions. It was observed that the ORF1 showed greater flexibility at the thumb and PAD as compared to finger and lower palm region. The ORF2 showed greater flexibility at thumb and lower palm while finger and PAD region showed lesser flexibility.

### Residue-residue interactions

**Figure 6:**
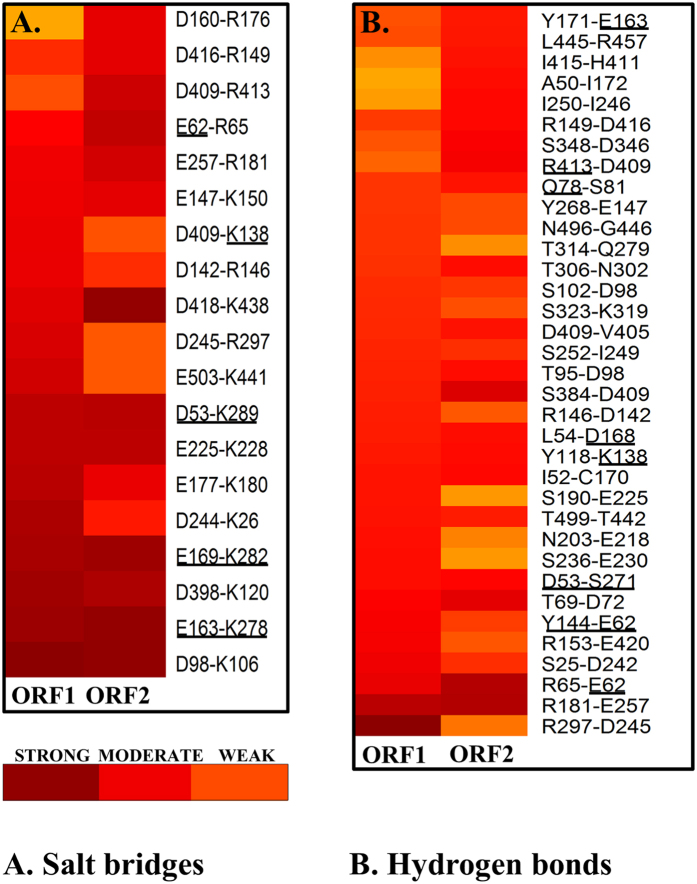
The heat map for salt-bridge and hydrogen bonding interactions. The panel (**A**) Salt bridges (**B**) Hydrogen bonds are shown across the two ORFs during the molecular dynamics. The conserved residues are underlined. The interactions are classified according to the occupancy and divided as strong–dark red colour (>80% occupancy), moderate–red colour (>50 and <80% occupancy) and weak–orange colour (<50% occupancy) interactions.

**Figure 7:**
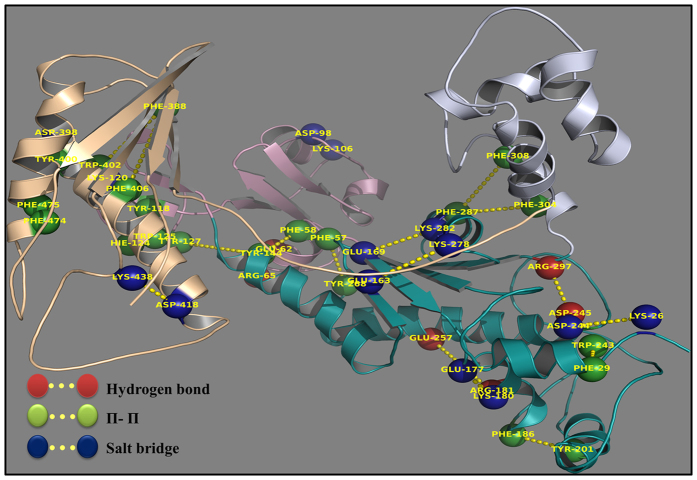
The network of interactions. The projection is a representation of highly consistent interactions (>80% occupancy) on the protein 3D structure. The plot shows residues participating in hydrogen bonds, π-π and salt bridges interactions in red, green and blue colour respectively. It also shows the chain of interactions (dotted lines) connecting different domains.

A conformational state of a protein is a manifestation of properly directed strong residue interactions. In the current study, an investigation has been made to get insight into residue-residue interactions that may play important role in stability and domain flexibility in the two ORFs. We examined the hydrogen bonds (Supplementary Fig. 9), the salt bridges and the π-π interactions in each of the snapshot of the last 60 ns trajectory. A heat map was generated to better understand the occurrence of hydrogen bond and salt bridge interactions throughout the trajectory (Fig. 6). Additionally, the interactions having >80% occupancy were mapped to protein 3D structure to visualize residue-residue interaction networks (Fig. 7).

#### Salt bridge interactions

**Figure 8:**
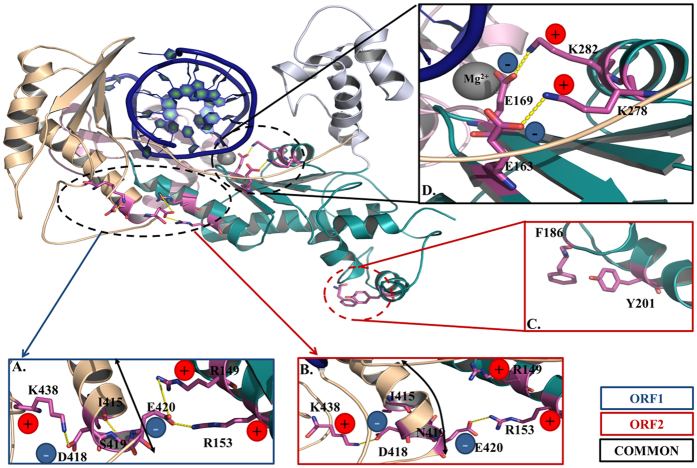
A closer view of residue interaction. The structure of CaPolƞ showing novel interactions and major differences in the ORFs, furthers the inserts (**A–D**) are shown separately. (**A**,**B**) Interactions of neighbouring residues of Ser419 in ORF1 or Asn419 in ORF2 causing a differential bending of helix at the PAD. (**C**) Interactions of neighbouring residues of Ala210 in ORF2 inducing a novel π-π interaction between F186–Y201. (**D**) A closer view of the molecular tetrad in both the system. The interaction between conserved residues [E163(β_7_)–K278(α_K_) and E169(β_8_)–K282(α_K_)] forming a molecular tetrad besides the catalytic triad is shown in the presence of Mg^2+^.

Interestingly, this study has revealed the presence of two parallel salt bridge interactions holding together the beta sheets β_7_ & β_8_ with the α_K_ helix. The residues E163 (in β_7_)–K278 (in α_K_) and E169 (in β_8_)–K282 (in α_K_) are involved in the formation of a molecular tetrad that is situated very close to the catalytic triad. The rectangular molecular tetrad appears to provide a platform which is stabilizing the spatial orientation of residues nearby catalytic center (Fig. 8). A detailed examination revealed that these four residues are also completely conserved across species (Fig. 1) indicating evolutionary relevance of these residues in the activity of Polη. Interestingly, the residue E169 also co-ordinates with one of the magnesium ions (Mg^2+^) and its spatial orientation can be crucial for the polymerase activity. Therefore, any mutation in E169 residue may result in loss of magnesium binding and collapse of the local structure^21^. The other possible salt bridges in palm domain of ORF1 are between the residues E177-K180 and D244-K26. Another interaction between residues D98-K106 at the tip of finger domain was found with high occupancy in both the ORFs. On the other hand, the interactions between D418-K438, E62-R65, and D409-R413 were found to be comparatively weaker in case of ORF1.

#### Hydrogen bond analysis

These intramolecular hydrogen bond interactions were plotted to compare between the two ORFs (Supplementary Fig. 9). It was found that the average number of hydrogen bonds for ORF1 and ORF2 were 90.80 and 85.09 respectively indicating that the ORF1 have 5 extra hydrogen bonds on average as compared with the ORF2. This may explain the higher conformational stability and rigidity of ORF1 as compared to ORF2. The analysis of hydrogen bond network led us to two significant observations (i) the residue interaction between R297-D245 (Fig. 6B) has high occupancy in ORF1 but has lesser occupancy in ORF2. Interestingly, this interaction is inter-helical (α_L_ and α_J_) and is present at the back side of thumb domain connecting with the lower palm domain. The lesser occupancy of this hydrogen bond interaction in ORF2 might have an important role in the observed high flexibility of thumb domain as compared to that of the ORF1 (Fig. 7). (ii) The residue interaction between R181 (α_G_)-E257 (α_j_) that makes an inter-bridging helix interaction at lower palm domain was found to be of highest occupancy for both ORFs. It is clear from that the occupancy of H-bonds and salt bridges is greater in ORF1 as compared to ORF2 which may be the reason for increased conformational stability.

#### π-π interaction analysis

The analysis of inter-residue π-π interactions indicated that a total of thirteen interactions are common among the two ORFs with slight differences in occupancy (Supplementary Table S2). However, some interactions e.g. the interaction Y201 (α_H_)-F186 (α_G_) and F29 (α_B_)-W243(α_J_) are absent in ORF1 as compared to ORF2. Interestingly, this analysis also revealed a network of high occupancy π-π interactions connecting different domains and probably responsible for the open right handed shape of Polη. These interactions together with hydrogen bond and salt bridge interactions may play an important role in the stability of the structure (Fig. 7).

### Estimation of DNA Binding energy

We have calculated the binding affinity of DNA with each ORF by Molecular mechanics with Generalized Born Surface Area continuum solvation approach (MM/GBSA) using Amber12. The bonded energy terms like bond, angle and dihedral were estimated using molecular mechanics. While the GB equation helps in the estimation of polar contributions, the surface area analysis helps in the finding the non-polar terms. In order to estimate the binding free energy of DNA, snapshots were taken at regular interval of 120 picoseconds from last 60 ns timescale (500 frames) from each system. In each snapshot, the water and ions were removed and GBSA was calculated. It is observed that the binding free energy of DNA in both the ORF complexes is not different significantly (−159.85 ± 49.44 kCal/mol in ORF1 and −129.33 ± 34.39 kCal/mol in ORF2, mean difference −30. 55 kCal/mol).

### *C. albicans* ORFs complement RAD30 function in *S. cerevisiae*

**Figure 9:**
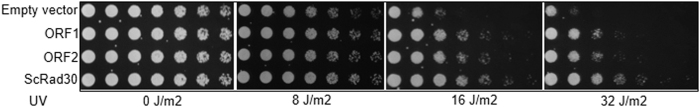
UV Sensitivity of *S. cerevisiae* rad30Δ strain. Cells of genomic RAD30 deletion yeast strains with empty vector or expressing RAD30 ORFs under a constitutive promoter from an overnight YPD culture were serially diluted, spotted onto YPD plates and subjected to different doses of UV as indicated. The plates were covered and incubated at 30 °C for 3 days and then photographed.

Extraordinary conservation of structures and similar positioning of multiple critical residues of both the *C. albicans* ORF*s* with ScRad30p instigated us to check their function in yeast. Deletion of RAD30 in yeast causes sensitivity to UV radiation. To test the functional significance of Candida ORFs, we examined the ability of these ORFs expressed under a yeast constitutive promoter to suppress UV sensitivity of *rad30Δ* strain. As shown in Fig. 9, unlike strain containing the empty vector, both the ORFs restored UV sensitivity of the *rad30Δ* strain to a level similar to that seen in the wild-type ScRad30. Thus, it suggests that these ORFs not only they share similar amino acid sequences and structures with other Polη, they are also functionally alike.

## Discussion

LOH or aneuploidy or point mutations in certain genes leads to genotypic difference resulting in phenotypic diversity in a species^10^. Interestingly, clinical isolates of *C. albicans* exhibit a considerable amount of natural heterozygocity and it is believed that high degree of heterozygosity in *C. albicans* might play an important role in microevolution of diversified species which might be required for its survival in various adverse conditions, and pathogenicity^9^,^11^,^12^,^32^,^33^,^34^,^35^. A genome-wide single-nucleotide polymorphism (SNP) map of SC5314 *Candida* strain recorded a total of 62534 high-confidence polymorphisms both in coding and noncoding sequences, and about 89% of the total are single-base substitutions^36^. The heterogeneity of *C. albicans* coding sequences has been poorly explored. The objective of this study was to identify a structural ortholog of ScPolη, determine the difference in dynamic behaviour of the two heterozygous ORFs in *Candida* Polη, and to understand possible structure-function analysis of the DNA polymerase by a comprehensive computational approach. By taking advantage of available 3D structures and using molecular simulation approach we have identified evolutionarily conserved residues, predicted the structure and function of *Candida* Polη as well as deciphered novel molecular interactions that might provide stability and flexibility to the structure.

### Evolutionary conservation in structure and function of finger and palm domains of Polη among eukaryotes

An amino acid comparison of ten Polη sequences across different species selected from fungi to worms, plants and animals revealed fourty one residues (~10% of the ORF) in the amino-terminal catalytic domain are identical including in the two ORFs of *C. albicans*. Interestingly majority of these residues are located in finger and palm domains that constitute the catalytic center of the enzyme suggesting that the mechanism of translesion DNA synthesis by Polη remains the same during evolution. All these identical residues are distributed in ten conserved patches of residues that also include earlier described motifs I-V. These five motifs and patches contain most (~90%) of the conserved residues. Although the functions of motifs I-V are known, the precise roles of other conserved patches are yet to be explored. For example role of residues like P73, Q78, K138, Y144, R145 and K386 are yet to be annotated. The residue Y144 is involved in a π-π interaction with F58 and this interaction stabilizes the finger. As these residues are also conserved, we predict similar interaction in other eukaryotic Polη as well. DNA polymerases discriminate between rNTPs and dNTPs; and F58 (F35 in yeast) of motif-I of Polη functions as a steric gate to prevent incorporation of rNMPs during DNA synthesis^27^. Genetic studies in yeast showed that strain expressing Polη with F35A mutation is inefficient in carrying out TLS and exhibits severe UV sensitivity. Biochemical studies have demonstrated the role of Q38 (Q78 in CaPolη), Y52 (Y87 in CaPolη), and R61 (R96 in CaPolη) of human Polη in governing polymerase fidelity as substitution to alanine causes both changes in fidelity depending upon the lesions in DNA substrate. As the model structure shows similar spatial positioning and orientation of these motif-II residues, similar key roles during TLS is expected^28^.

The high resolution crystal structures of yeast Polη suggest that D30, D155 and D156 amino acids in palm domain catalyze the nucleotidyl transfer reaction; therefore substitution of these residues to alanine makes the polymerase inactive. The incoming dNTP is aligned and stabilized by hydrogen bonding with Y64 and R67 from the fingers domain and K279 from the palm domain, and its sugar packs against the aromatic ring of F35. Our model structure predicts D53, D168, E169, Y87, R90, and F58 are the equivalent *Candida* Polη residues^26^ and therefore it was not surprising to notice suppression of UV sensitivity by these ORFs of yeast *rad30* deletion strain. Mutational inactivation of D168A, E169A in *C. albicans* Polη did not complement UV sensitivity in *rad30Δ* strain of *S. cerevisiae* (our unpublished observation).

### Novel molecular interactions might play a crucial role in DNA synthesis by Polη

In addition to molecular interactions as predicted in X-ray crystal structures, our study has predicted two major novel interaction networks in Polη (1) a series of π-π interactions spanning from thumb to PAD through the finger and (2) a molecular tetrad in the palm. The π-π network is appeared to form the backbone of open right handed polymerase structure. The PAD is enriched with π-π interactions. Although PAD is least conserved domain, it is essential for DNA polymerase activity. It has been demonstrated that the β-12 (amino acids 316–325 in human and 379–388 in *Candida* ORFs) stands parallel to the DNA template and makes necessary contacts to correctly place the template strand with the catalytic core of the enzyme^37^. Hydrogen bonds involving conserved T318 (S381), K317 (K380), P316 (V379) and R361 (E423) with DNA template phosphate residues are critical for the interaction as mutation of these residues to alanine reduces polymerase activity drastically. The R361S SNP has been identified in a XPV patient suggesting the importance of this interaction^38^. We believe that loss of π-π interaction in PAD will destabilize DNA-PAD interaction. Among these, the π-π interactions between W402 with F388 appear to be crucial; and to our surprise these residues are almost identical across the species.

Mutational analysis of invariant acidic residues such as D30, E39, E79, D155, E156, D160 and D235 in yeast Polη has been carried out^26^. Among these, D30, E39, and D155 residues are found to be essential for Rad30 activity and function; and the E156 is not essential for activity but it greatly affects the efficiency of nucleotide incorporation. Structural analyses suggest that D30 and D155 bind to metal ions for nucleotidyl transfer reaction. This study has revealed presence of a molecular tetrad involving residues E163 (E141 in yeast)-K278 (K258 in yeast) and E169 (E156 in yeast)-K282 (K272 in yeast) situated very close to the catalytic triad. Interestingly all the residues involved in the pattern formation are completely conserved and the interactions between them has also shown occupancy >90%. The molecular tetrad appears to provide a platform which is stabilizing the spatial orientation of residues nearby catalytic center (Fig. 8D). Absence of this tetrad could result in destabilization of the primer template alignment and possibly the movement of finger as well. It is not surprising to observe inefficient and error prone DNA synthesis by E156A mutant of ScRad30.

### Heterozygous Candida Polη ORFs exhibit subtle differences in the structure

The overall structure of the ORFs appears to be similar despite substitution of three residues in the ORFs (D70N, T218A and S419N) possibly due to retention of majority of the molecular interactions including the π-π network, molecular triad and tetrad. The two ORFs have shown similar flexibility in the finger domain, and comparison to other domains, finger is the least flexible. Similar results were also observed by Ucisik *et al*. where they found the finger domain in Polη to be least flexible among other domains^39^. A comparison of RMSD between the ORFs and *S. cerevisiae* revealed that all of them have almost similar structural stability, however, the ORF1 showed an RMSD profile that is closer to that of the *S. cerevisiae* (Supplementary Fig. 2). Similarly, RMSF analysis revealed a comparable flexibility pattern for ORF1 and *S. cerevisiae* Polη (Supplementary Fig. 10). This suggests that, among the two ORFs, the dynamic behaviour of ORF1 is closer to *S. cerevisiae*, and inspires for further characterization.

The RMSF analysis further revealed a notable difference in the flexibility at the palm domain (α_K_) among the two ORFs probably due to the extra stabilization by Thr at 210 position that might be involved in formation of hydrogen bond interactions with the neighbouring residues of ORF1 or ORF2. However, a closer analysis of the MD trajectory did not reveal any such interaction in ORF1 rather the hydrophobic residues which are in immediate vicinity of the Ala210 side chain of ORF2 may play a major role. The Ala210 side chain interacts with the neighbouring Leu213 side chain and that exerts a pulling force on the helix α_K._ In turn it places the Tyr201 in position for a π-π interaction with Phe186. The analysis also revealed that the residues at the thumb, and PAD domain in ORF2 showed significant variations in dynamic behaviour as compared to ORF1. The difference in the flexibility of the thumb domain among the two ORFs may be attributed to the absence of inter-helical hydrogen bond interaction between R297 (α_L_)-D245 (α_J_) in ORF2. Interestingly, lesser flexibility at the PAD region in ORF2 resulted from the substitution of S419N at the end of α_R_ region. The substitution of Ser by bulkier Asn results in slight change in helix conformation. As a result the interaction between D418 (α_R_)- K438(loop) becomes stronger as indicated by increase in the occupancy. Also, there is loss of interactions between E420 (α_R_)-R149 (α_F_) and weaker interaction between E420 (α_R_): R153 (α_F_). This results in slightly bending up the loop and helix (α_R_) (Fig. 8).

### Perspective

The conclusions derived from MD trajectories suggest that the structure and dynamics of the ORFs domains showed no significant differences except at the thumb and lower palm domain. Although both the ORFs equally complement RAD30 function of *S. cerevisiae* and are the functional homologues, it is intriguing to see the impact of such differences in the DNA polymerase and lesion bypass activities. A major finding of this study is the identification of a conserved tetrad, which may play a crucial role in the activity of the Polη. Stress induced mitotic recombination or gene conversion can result in LOH for certain chromosomal regions and that can result in new phenotypes like fungal drug resistance, morphology etc.^10^,^32^,^40^. Both the biological rational and the significance of the allelic heterogeneity of Polη in pathogenesis of *C. albicans* will be explored to consider Polη as a fungal drug target.

## Materials and Methods

### The protein structure modeling

The Polη structures encoded by ORF1 and ORF2 were modeled. The protein sequences were obtained from NCBI [accession ID: gi|68469958 (ORF1) and gi|68469717 (ORF2)]. A blast^41^ search was done, and the X-ray crystal structure of *S. cerevisiae* Polη (PDB ID: 2WTF) was found suitable as a template based on significant identity (41%), query coverage (62%) and e-value (ORF1: 2e-99, ORF2: 9e-100)^42^. The sequence alignment of Polη ORFs with the crystal structure of *S. cerevisiae* was performed using PROMALS 3D web server^43^. PROMALS 3D uses progressive multiple alignment approach to perform the sequence alignment of protein sequences. The generated sequence alignment was used for the Polη structure modeling using Modeller 9.16 software^44^. The modeller generates protein models by satisfaction of spatial restraints, which can be further refined by using molecular mechanic or dynamic methods. It generates a molecular probability density function (molpdf) score, which is used to rank the models. In the current work, ten models were generated for *C. albicans* Polη using ligand supported mode i.e. with DNA positioned in the modeled structure. The best homology model was selected with the lowest molpdf score.

Finally, the generated models were checked for stereochemical quality using Ramachandran plot^45^. It was noted that a majority of residues (ORF1–99.60%, ORF2–99.60%) were found in the allowed regions (Supplementary Fig. 11). As stated earlier, the two ORFs differ by only three amino acids; the residue (D_ORF1_ → N_ORF2_) is located between the interconnecting loop α_C_ and β_2_ at the 70^th^ position in thumb region, the residue (T_ORF1_ → A_ORF2_) is located at 210^th^ position in palm region (α_H_) and the residue (S_ORF1_ → N_ORF2_) is located at the 419^th^ position in PAD region (α_R_).

### System setup and MD simulation

In the current study, the preliminary topology and coordinates for Polη protein and DNA complex were generated using tleap module of AMBER 12.0^46^ while all other hetero atoms were removed. Each Polη complex was solvated in a rectangular water box (TIP3P water model) with a buffering distance of 10 Å. A total of 18 Na^+^ ions were added to ensure electro-neutrality of the solvated system. The final system had 24,598 and 23,249 water molecules for ORF1 and ORF2 respectively. These water molecules were treated using SETTLE algorithm^47^. The associated system topology and coordinates were generated by applying ff99SB force field parameters for MD simulation^48^. The MD simulations were executed using NAMD software that is known for its efficient parallel computation^49^. In the present study, most of our simulations were executed over 100 processor cores. Prior to the simulation, the system was properly minimized with a stepwise minimization protocol. Firstly, the water molecules and ions were minimized which was then followed by hydrogen atoms and the side chains of the complex. The side chains were minimized for 40000 steps while the backbone atoms and the bond lengths of hydrogen atoms were kept fixed. Thereafter, all the atoms were allowed to relax freely and the whole system was energy-minimized for 40000 steps with nominal restraints on C-alpha atoms and DNA backbone atoms (10 kcal/mol) to prevent any abrupt change in structure. Subsequently, an equilibration protocol was followed where the system was heated gradually from 0–310 K in steps of 30 K with a canonical ensemble (NVT). At each step, a 20 picosecond (ps) simulation was run to allow the system to adjust to the temperature. Once the system attained 310 K, an isobaric and isothermic ensemble (NPT) was applied for a period of 100 ps with a constant pressure of 1.0 bar using Langevin dynamics^50^. Finally, the applied restraints on C-alpha atoms and DNA were removed and the system was equilibrated for 1 ns at 310 K using Langevin piston coupling algorithm. During the whole simulation, the Particle Mesh Ewald sum algorithm (PME) algorithm was used to calculate the long-range electrostatic interactions with fixed periodic boundary conditions^51^. The covalent-bond interactions involving hydrogen bonds were constrained using SHAKE algorithm^52^. Once the system simulated with constant 310 K temperature and 1.0 bar pressure than the production run was done for a time period of 80 ns. During the whole simulation time, a time step of 2fs was applied. The analyses of the MD trajectories were performed to get an insight on the structure and dynamic behaviour of two different ORFs of Polη. The trajectories were analyzed for RMSD, RMSF, Rg, SASA, PCA, CA and residue interaction networks etc. to understand various conformational changes and motions of secondary structures. These analyses were performed using Ptraj, VMD, MMTSB tool set and in-house Perl & Tcl scripts^53^,^54^,^55^.

### Multivariate statistical analysis

To understand the structural dynamics over the simulation time, we have subjected the trajectory to PCA^56^. It is a widely used statistical tool for the analysis of unsupervised data. It clusters data points based on the distance, where data points with similar properties are grouped together and smaller sets are formed. In an MD simulation, the conformational space of the system throughout the trajectory can be linearly transformed to generate 3 N × 3 N covariance matrix. The eigenvectors can be derived from these directed cartesian coordinates and can be projected on time *vs* component axis. Several principal components can be used to derive a model, but the model that represents the most variance with least number of principal components is generally selected to retain maximum information with minimal noise.

We have used *K*-means clustering which is a powerful clustering technique to generate representative clusters for each trajectory^57^. It uses a linear square fit method to identify centroids by optimizing the squared deviation errors. These clusters can be plotted against RMSD values. In the current study, last 60 ns trajectory for each of the complex was used for extraction of PCAs using Ptraj software. The covariance matrix was constructed to generate eigenvalues for each frame. Thereafter, two principal components corresponding to eigenvalues of each frame were obtained. To identify native structures from each cluster over the time period, we have performed CA analysis. Initially, at 2 ps interval the snapshots from each trajectory were retrieved. Thereafter, these snapshots were used for CA analysis using MMTSB tool set. The *K*-means clustering was done with an RMSD (C_α_) gradient of 2 Å for a total of 30000 frames. Five different clusters were obtained and centroids for each of them were calculated. The centroid of the biggest cluster for each of the ORF was used for further analysis.

### Residue network mapping

The residue interaction networks are important for protein’s secondary structure maintenance, structural rigidity and functionality. In order to identify crucial interactions, we have calculated hydrogen bonds, the salt-bridges and the π-π interactions that are present in each frame during the simulation using VMD. The salt bridge interaction usually occurs when the amino acid containing anionic carboxylate (RCOO^−^) and the cationic ammonium (RNH_3_^+^) group forms both hydrogen bond and electrostatic interactions simultaneously with the same residue. A salt-bridge interaction was measured when the residues pairs are within the distance of 3.2 Å. Likewise, the hydrogen bond interaction is calculated between the polar hydrogen atom and a nearby (<3 Å) acceptor atom.

Similarly, the π-π interactions were calculated when one π electron cloud system attracts another nearby π electron cloud system. These interactions are pivotal in biological events such as molecular recognition. In this study, to identify the pair of π-π interacting residues, each hydrophobic ring centroid was considered to measure the inter-residue distance. In four aromatic residues i.e. Phenylalanine (Phe), Tyrosine (Tyr), Histidine (His) and Tryptophan (Trp); the six-membered carbon ring from both Phe and Tyr, five-membered ring in the indole group from Trp and imidazole group from His were used to calculate the ring centroids and plane angles. The residue pairs within a ring centroid distance of 4.5–7.0 Å (min-max) were considered in each frame for both the trajectories^58^. Further, for these residues normal vectors were calculated to identify the angle (θ) between the two planes. These interactions gave rise to two different structural geometries i.e. face-to-face (parallel) and T-shaped (perpendicular) interaction. If the geometrical angle between the planes of two rings was found to be 30° > θ > 150°, the rings were defined in face-to-face interaction; if the angle is between 30° < θ < 150°, the rings were defined in T-shaped (edge to face or perpendicular) interaction^59^. But, to map critically important residues, we filtered out those interactions that have less than 50% occupancy during the simulation. The remaining interactions were considered for further analysis. Moreover, if an interaction is having >50% occupancy in one ORF, it was plotted for both regardless of its occupancy in the other. Interestingly, all the observed π-π interactions in the current studies were found to be in T-shape^58^. These interactions were calculated for each frame of the trajectory using in-house Tcl and Perl scripts.

### Generation of *C. albicans* RAD30 constructs for functional analysis in *S. cerevisiae*

To clone putative DNA polymerase eta ORFs, a 50 μl PCR reaction mixture of 100 ng of *C. albicans* SC5314 genomic DNA, 250 μM dNTPs, 1 × Pfx enzyme buffer, 1.5 mM Mg2SO4, 2U of Pfx DNA polymerase (Invitrogen), and 20pmol each primers NAP03 (fp 5′-CCG GAA GCT TGG ATC CAC ATA TGT CCG TGA AAC AGG AAA CAC-3′) and NAP04 (rp 5′-GGC CGG TAC CGG ATC CTT AAT GAT TAT TTA GTT TG-3′) was thermo-cycled in a PCR. PCR condition included initial heating at 95 °C for 3′ followed by 34 cycles at 95 °C for 30 s, 55 °C for 45 s, and 68 °C for 2′. About 1.9 KB amplified PCR product was purified, digested with HindIII-Asp718 and cloned into similar sites in pUC19. While sequencing we observed two different orfs of RAD30 in SC5314 strain that corresponds to CaO19.866 (ORF1) and CaO19.8485 (ORF2). For complementation analysis in *S. cerevisiae*, BamHI fragments of these ORFs were subcloned into BamHI site of a 2μ based yeast vector with ADH1 promoter^60^. Theses constructs were transformed to *EMY74.7 rad30Δ* yeast strain for functional analysis. An empty YEplac181-ScADH1p (LEU2) or containing ScRad30 were also transformed to use as controls. The transformants were grown in SC medium lacking leucine (SC-leu) to maintain selection for the plasmid. When cultures had reached the midlogarithmic phase, they were washed by centrifugation, subjected to vertexing to disperse cell clumps, pelleted by centrifugation, and resuspended to a density of 2 × 10^8^ cells per ml. Appropriate dilutions of cells were spotted on YPD + Agar plate. UV irradiation was done at a dose rate of 8 J/m2/s. Following UV irradiation, plates were incubated in the dark, and incubated at 30 °C for 3 days and then photographed.

## Additional Information

**How to cite this article:** Satpati, S. *et al*. Comparative molecular dynamics studies of heterozygous open reading frames of DNA polymerase eta (η) in pathogenic yeast *Candida albicans.* *Sci. Rep.* **7**, 41087; doi: 10.1038/srep41087 (2017).

**Publisher's note:** Springer Nature remains neutral with regard to jurisdictional claims in published maps and institutional affiliations.

## Supplementary Material

Supplementary Information
