# Supplementary material for: Comparative molecular dynamics studies of heterozygous open reading frames of DNA polymerase eta (η) in pathogenic yeast Candida albicans
Source: Sci Rep. 2017 Jan 25;7:41087. doi: 10.1038/srep41087 (PMC5264235; doi:10.1038/srep41087)
Supplement: Supplementary Information [file srep41087-s1.doc]

**Supplementary information**

**Comparative molecular dynamics studies of heterozygous open reading frames of DNA polymerase eta (η) in pathogenic yeast *Candida albicans***

**Suresh Satpati^#^, Kodavati Manohar^$^, Narottam Acharya^$*^ and Anshuman Dixit^#*^**

**^#^**Department of translational research and technology development, Computational Biology and Bioinformatics laboratory, Institute of Life Sciences, Bhubaneswar-751023, Odisha, India.

**^$^**Department of Infectious Disease Biology, L**aboratory of Genomic instability and Diseases**, Institute of Life Sciences, Bhubaneswar-751023, Odisha, India.

**^*^**Corresponding authors

Anshuman Dixit, Institute of Life Sciences, Nalco Square, Bhubaneswar-751023, Odisha, India

Email: anshumandixit@gmail.com, anshuman@ils.res.in, Phone: +91674-2300137/2301476 ext. 281, Fax: +91674-230072

Narottam Acharya, Institute of Life Sciences, Bhubaneswar 751023, India Email: narottam_acharya@ils.res.in, Phone: +91674-2300137/2301476 ext. 278, Fax: +91674-230072

**
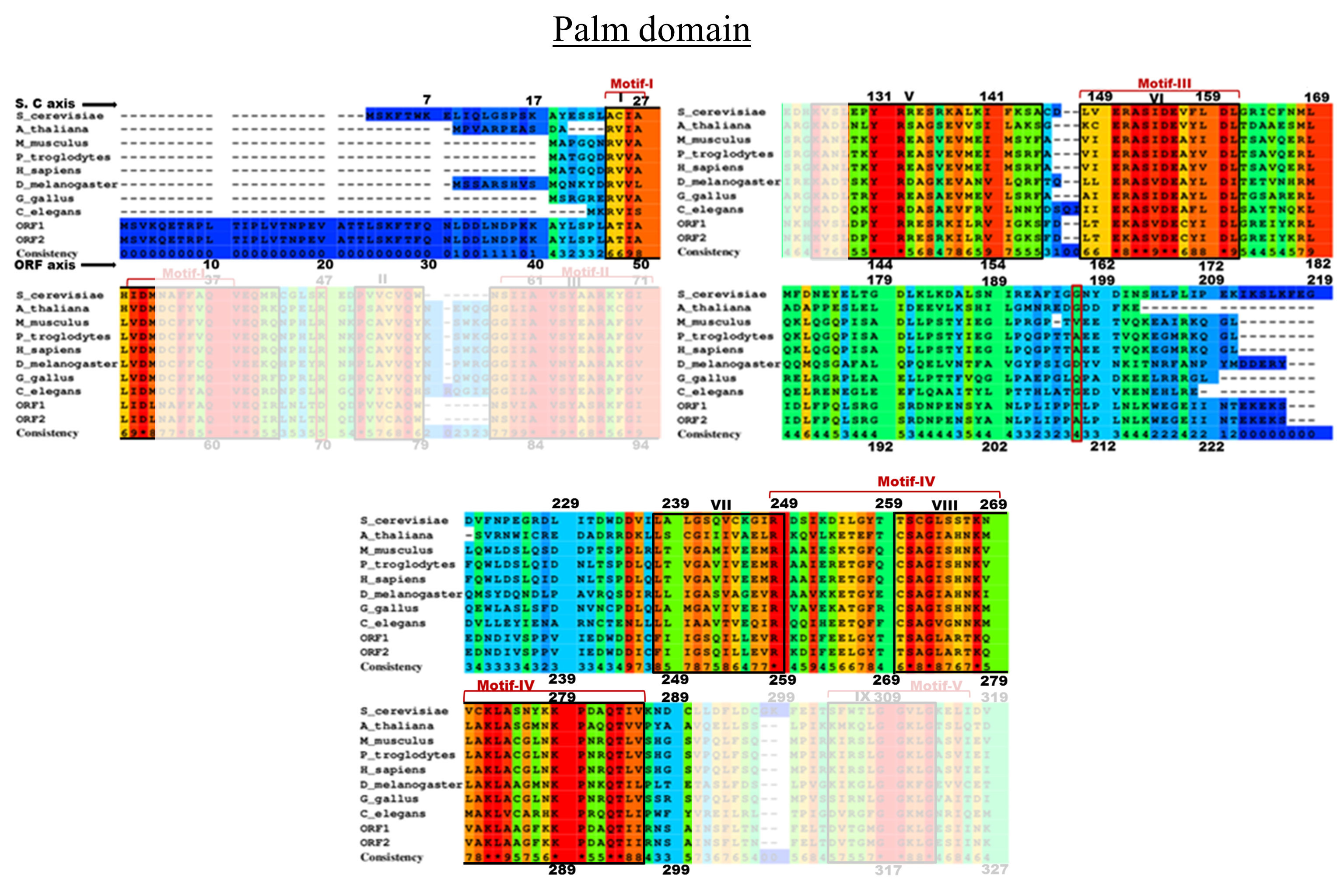
Supplementary Figures**

**A.**

***
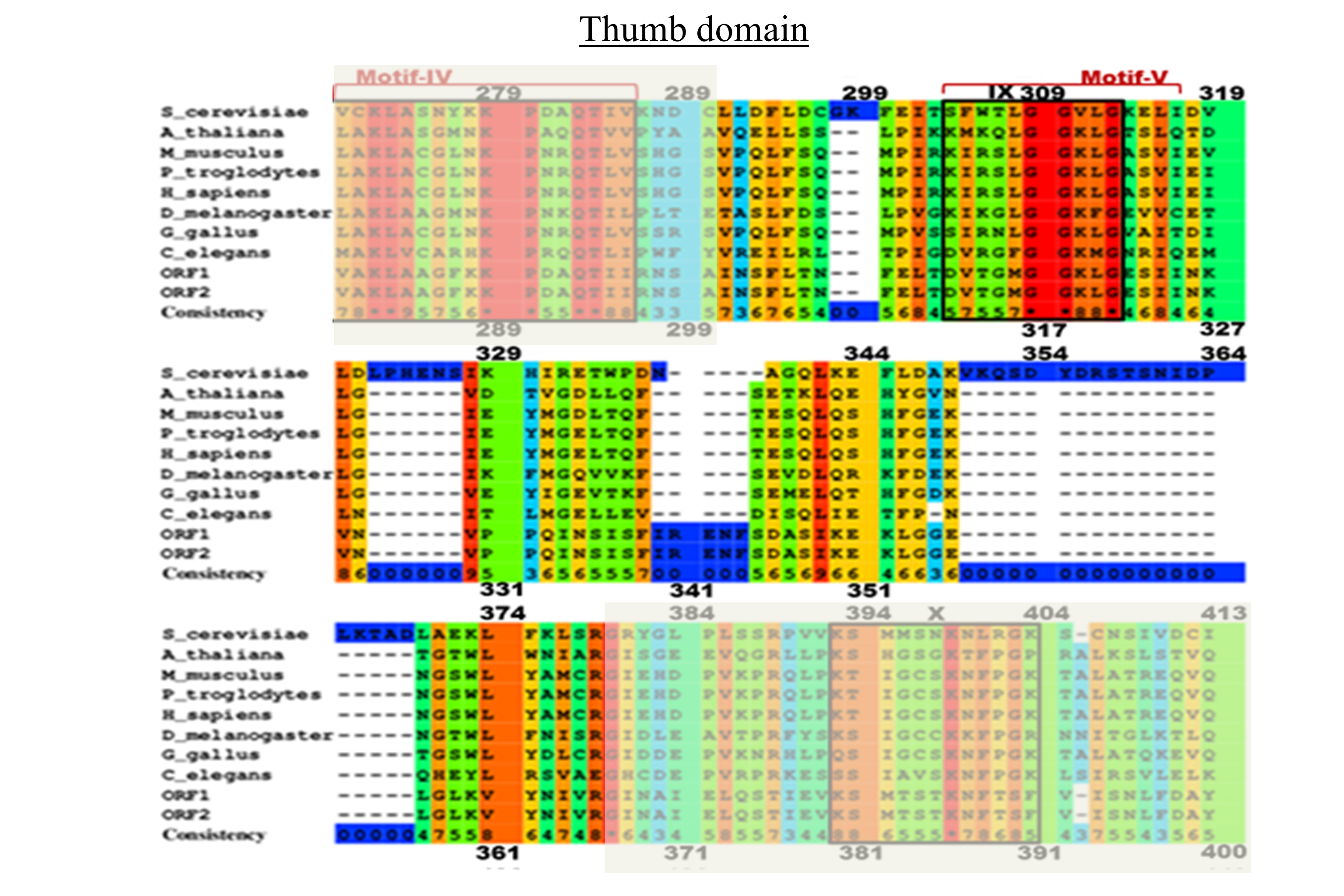

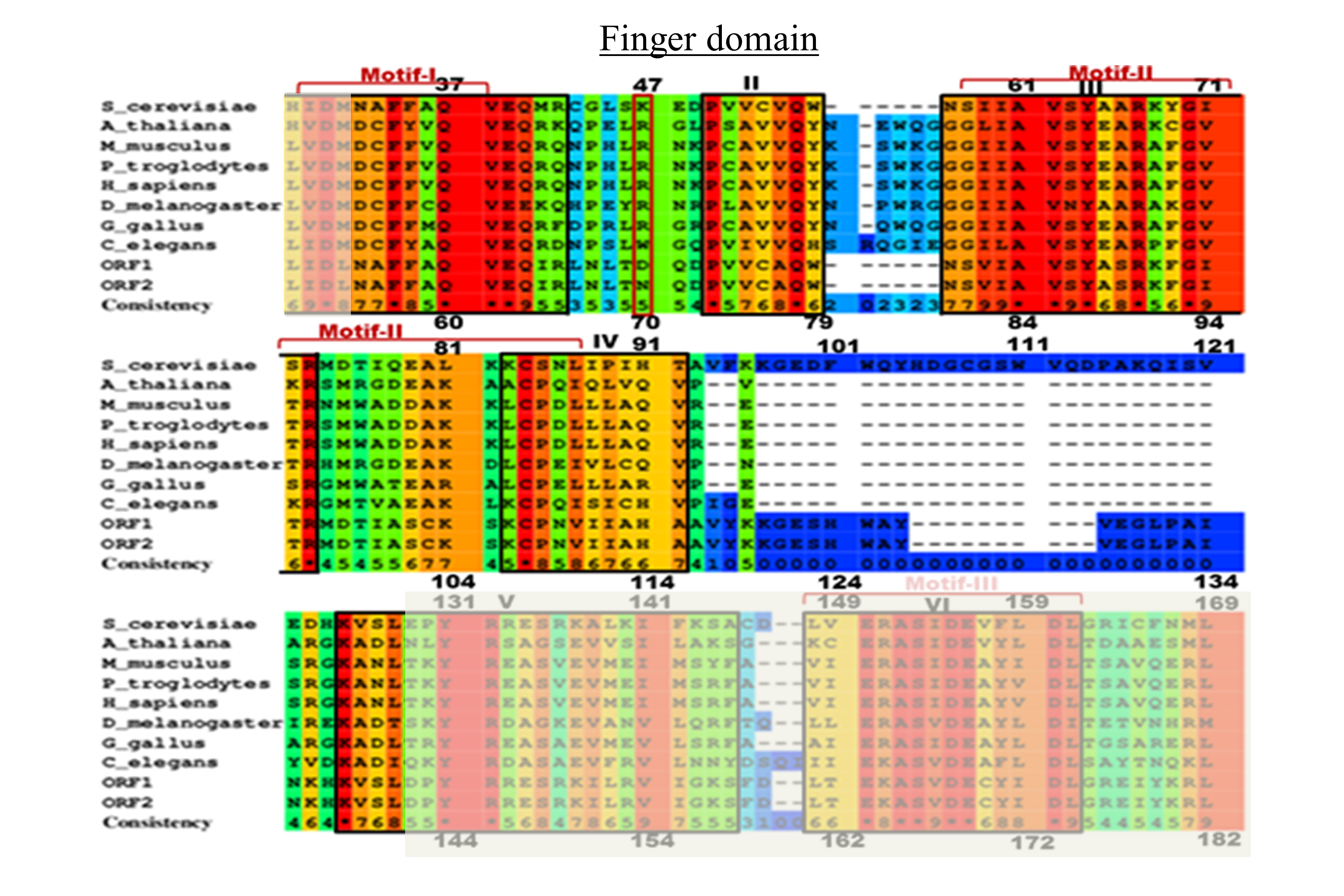
***

**B.**

**C.**

***
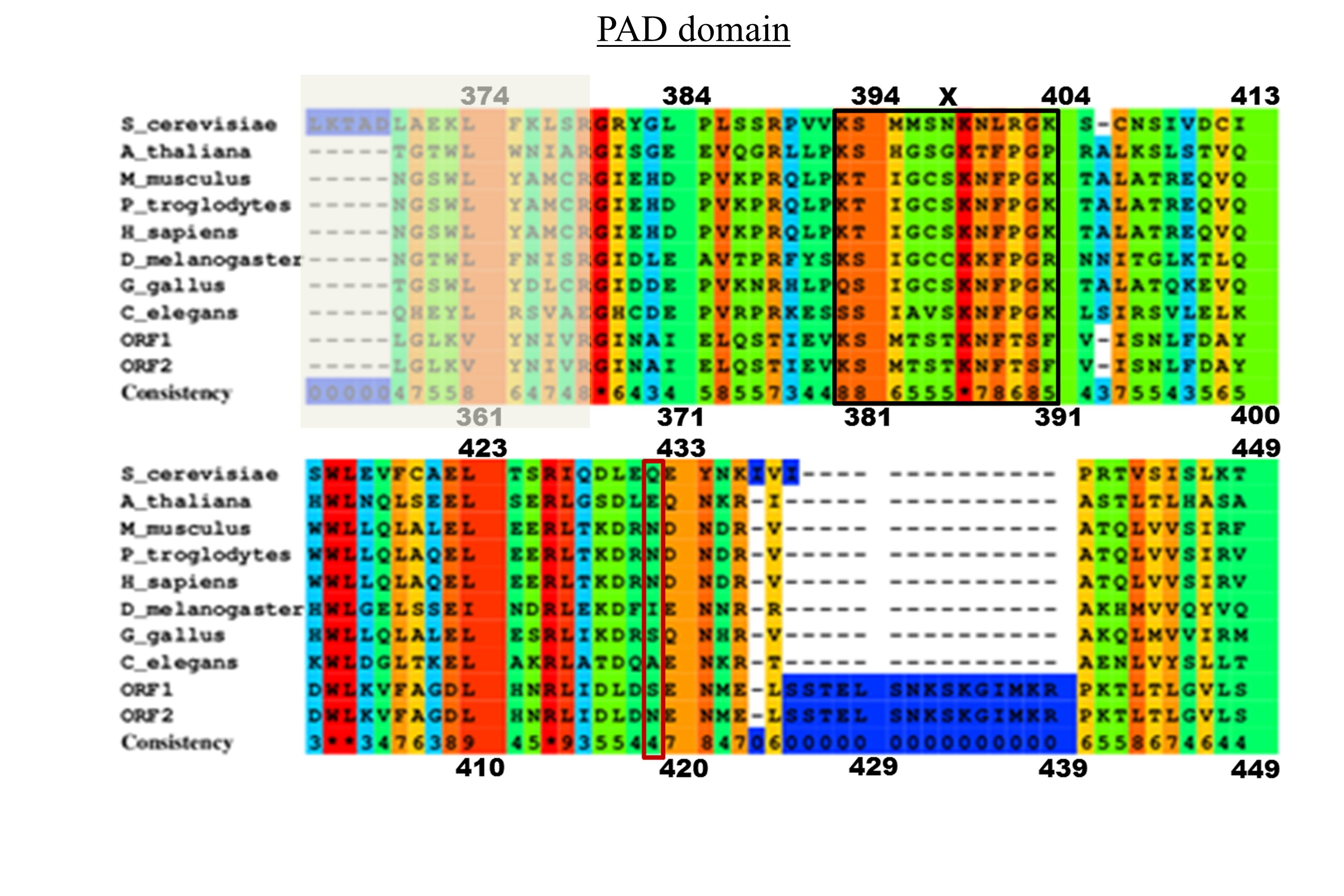
Supplementary Fig. 1(A-D):*** The multiple sequence alignment: The domain wise representation of conserved residues, motifs and identified patches in each species.

**D.**

**
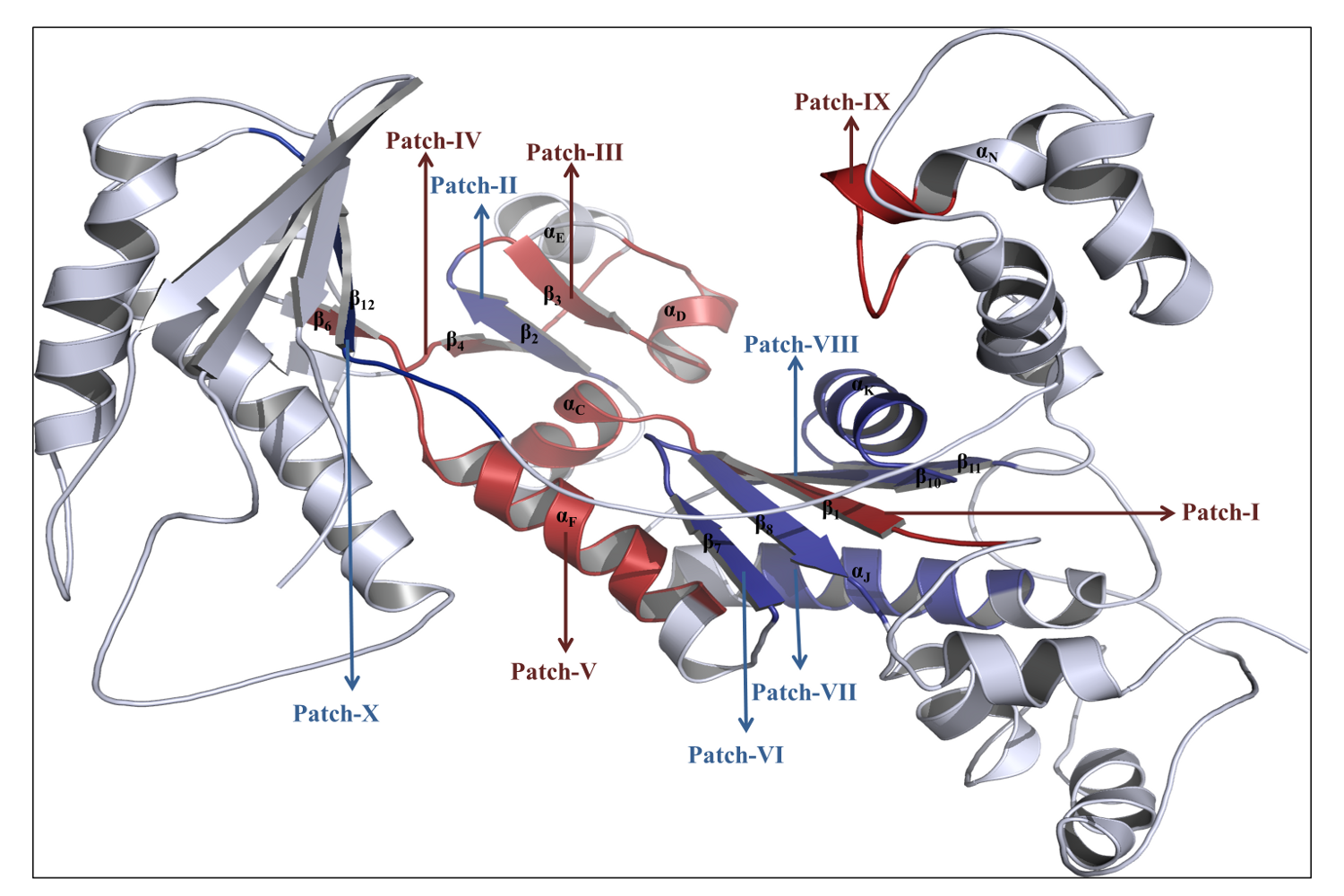
**

***Supplementary Fig. 1E:*** The three dimensional representation of identified patches on the modeled ORF structure. For easy identification patches on protein 3D structure are shown in blue and red colour.


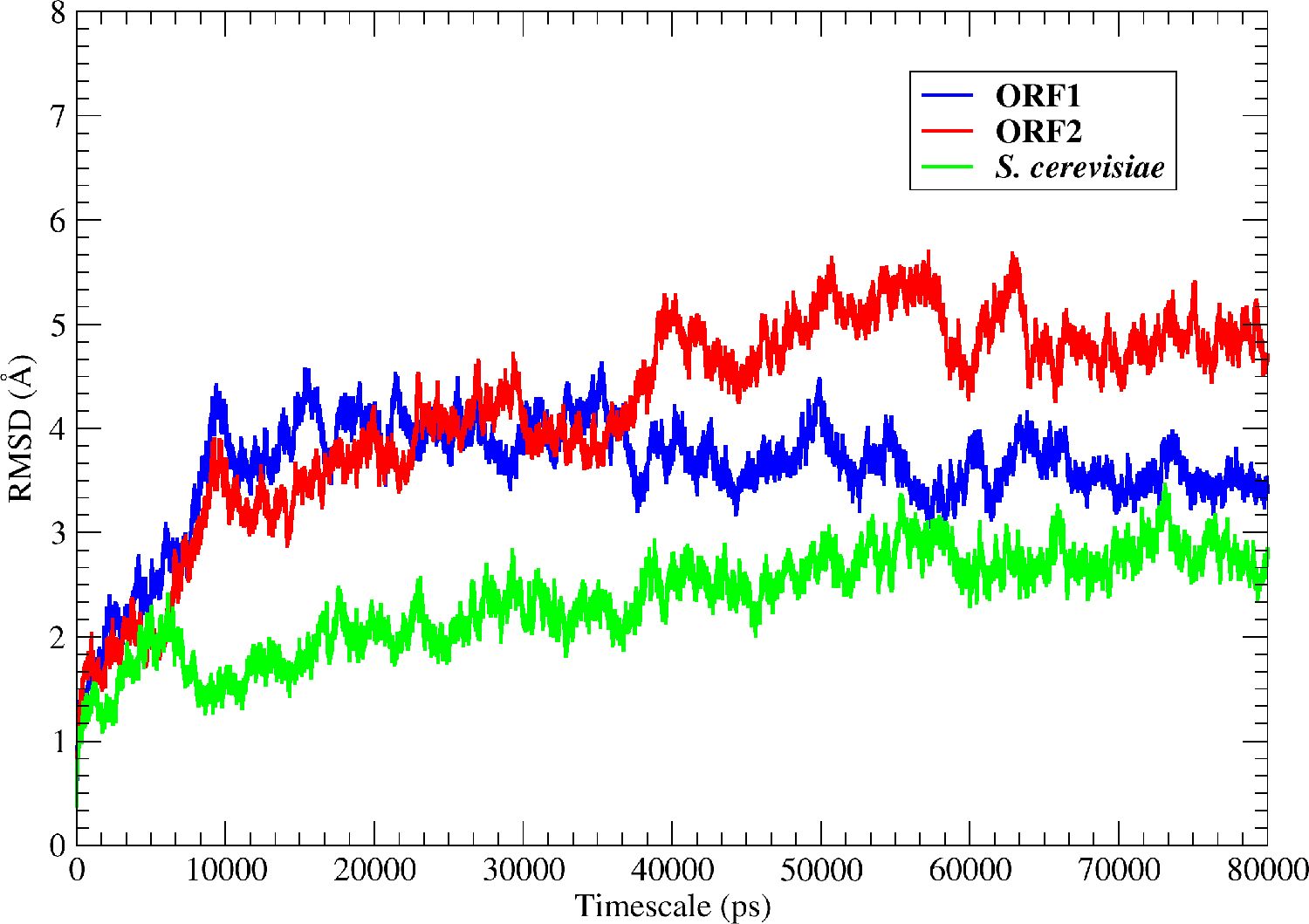
***Supplementary Fig. 2:*** The RMSD graph corresponds to the change in RMSD values between the three system trajectories during the entire run. The total run time (ps) and the RMSD (Å) are shown on ‘X’ and ‘Y’ axis respectively.


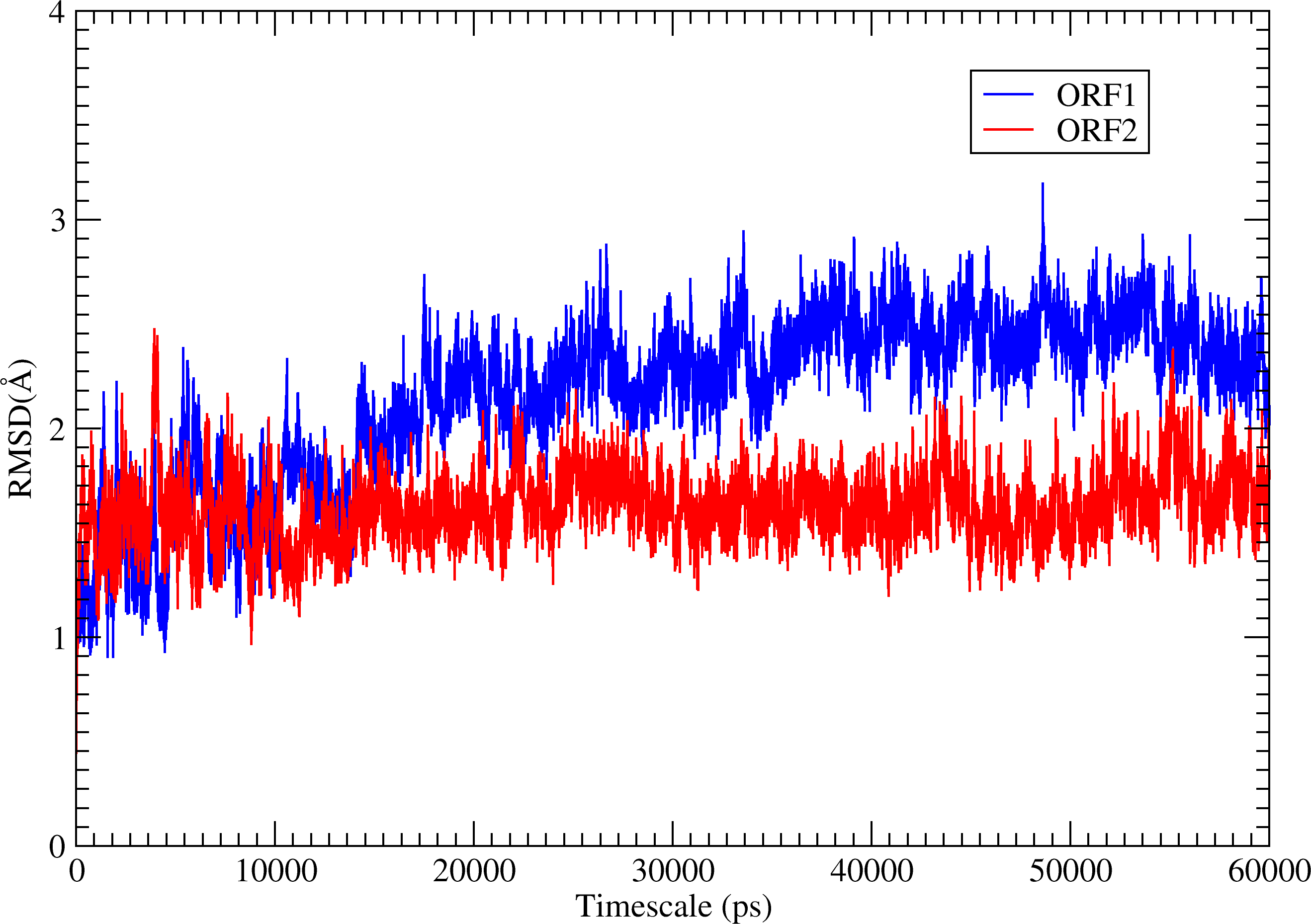


***Supplementary Fig. 3:*** The backbone RMSD plot for the DNA structural stability. The ‘X’-axis indicates the total frames from last 60 ns, while the change in RMSD value is shown on ‘Y’- axis. The RMSD for ORF1 and ORF2 are shown in blue and red lines.


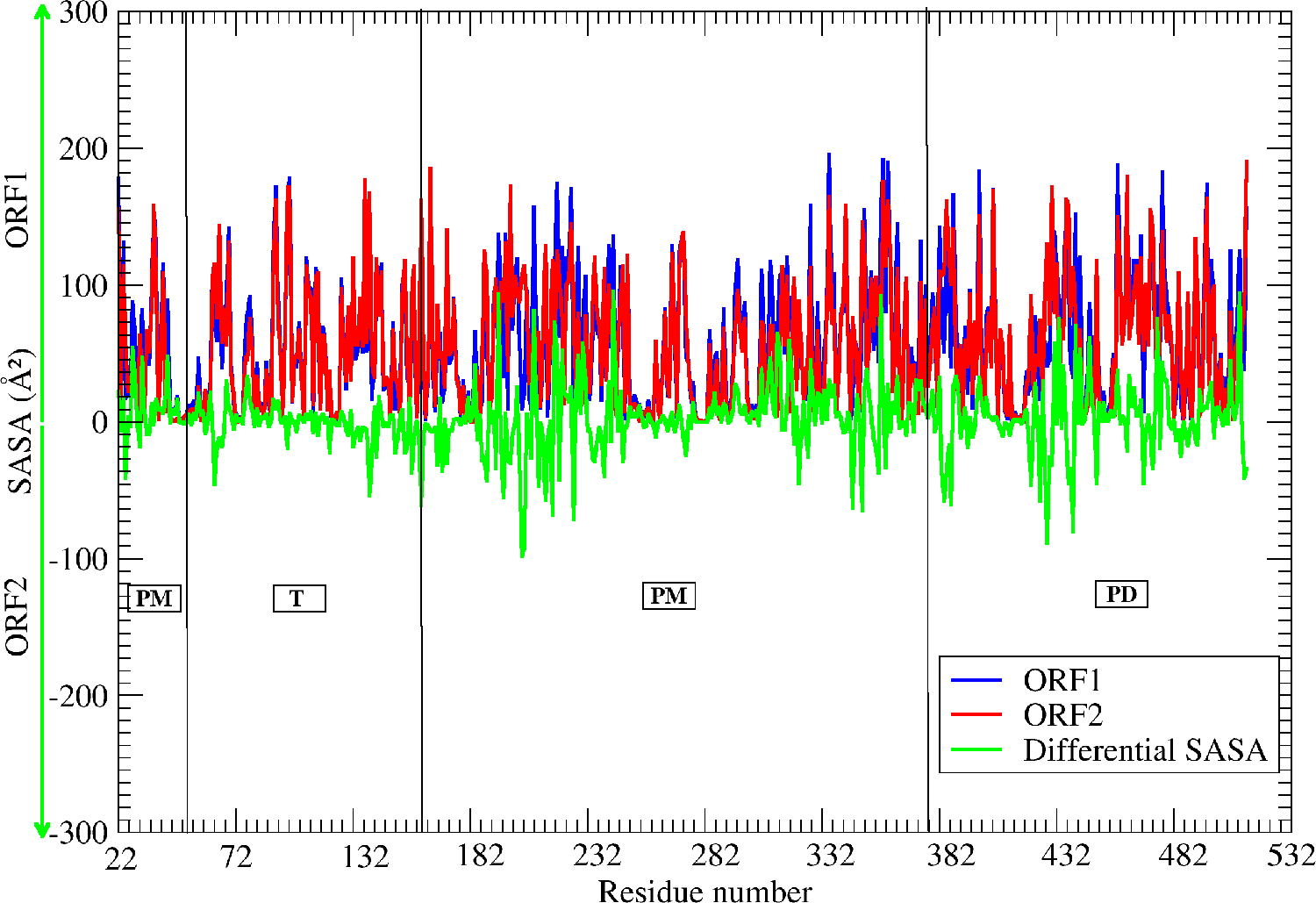
***Supplementary Fig. 4:*** The solvent-accessible surface area (SASA) plot depicts the SASA averaged over the simulation time for individual residues. The residue number are given on ‘X’-axis, while the change in SASA value is shown on ‘Y’- axis. The SASA for ORF1 and ORF2 are shown in blue and red lines. The green line represents the differential SASA (ORF1-ORF2) values.


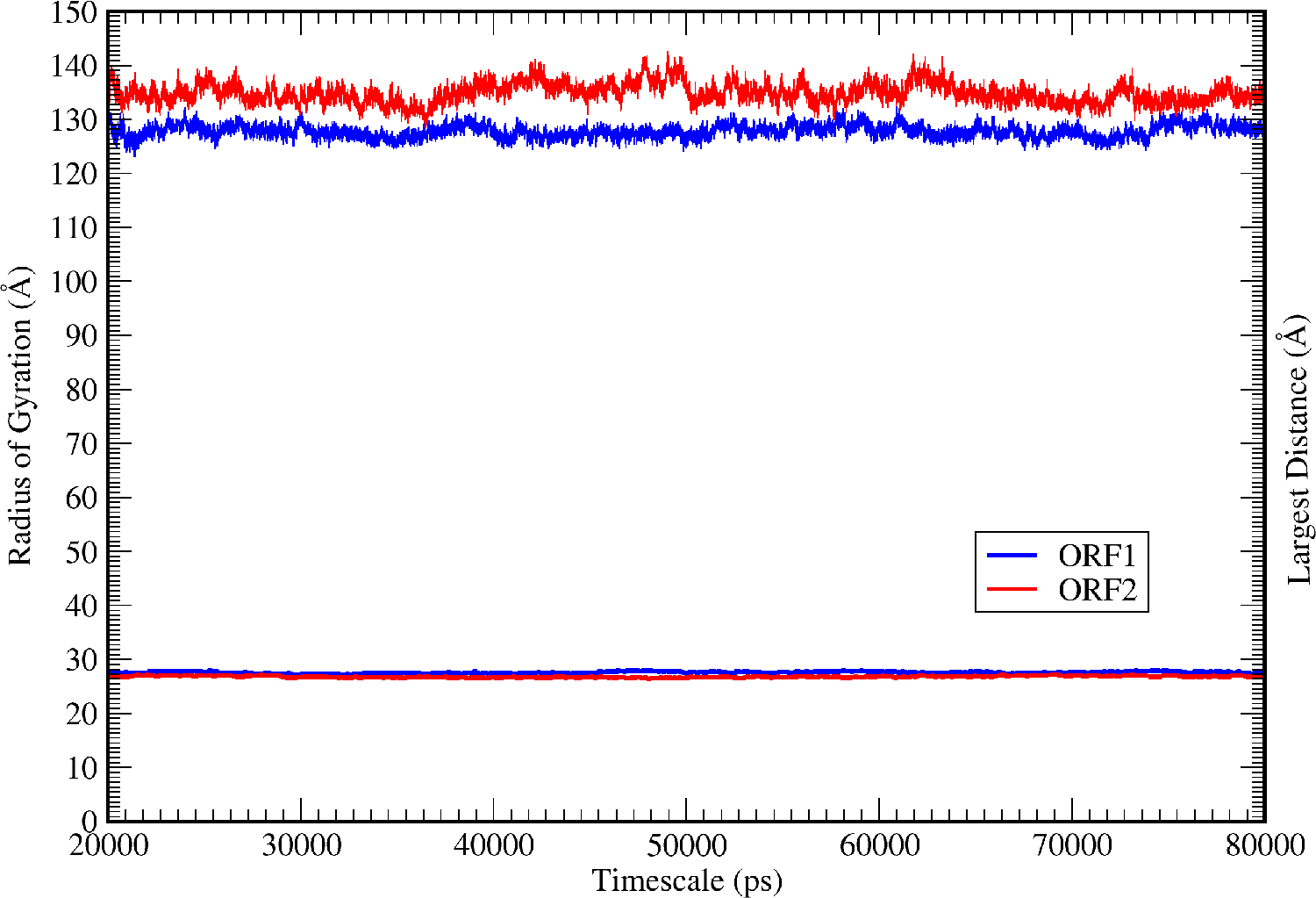


***Supplementary Fig. 5:*** The radius of gyration (Rg) depicts the protein compactness in each frame for the individual ORFs during simulation. The upper curves represent the largest distance between any two residues in each system. The lower curves show the radius of gyration during the simulation.


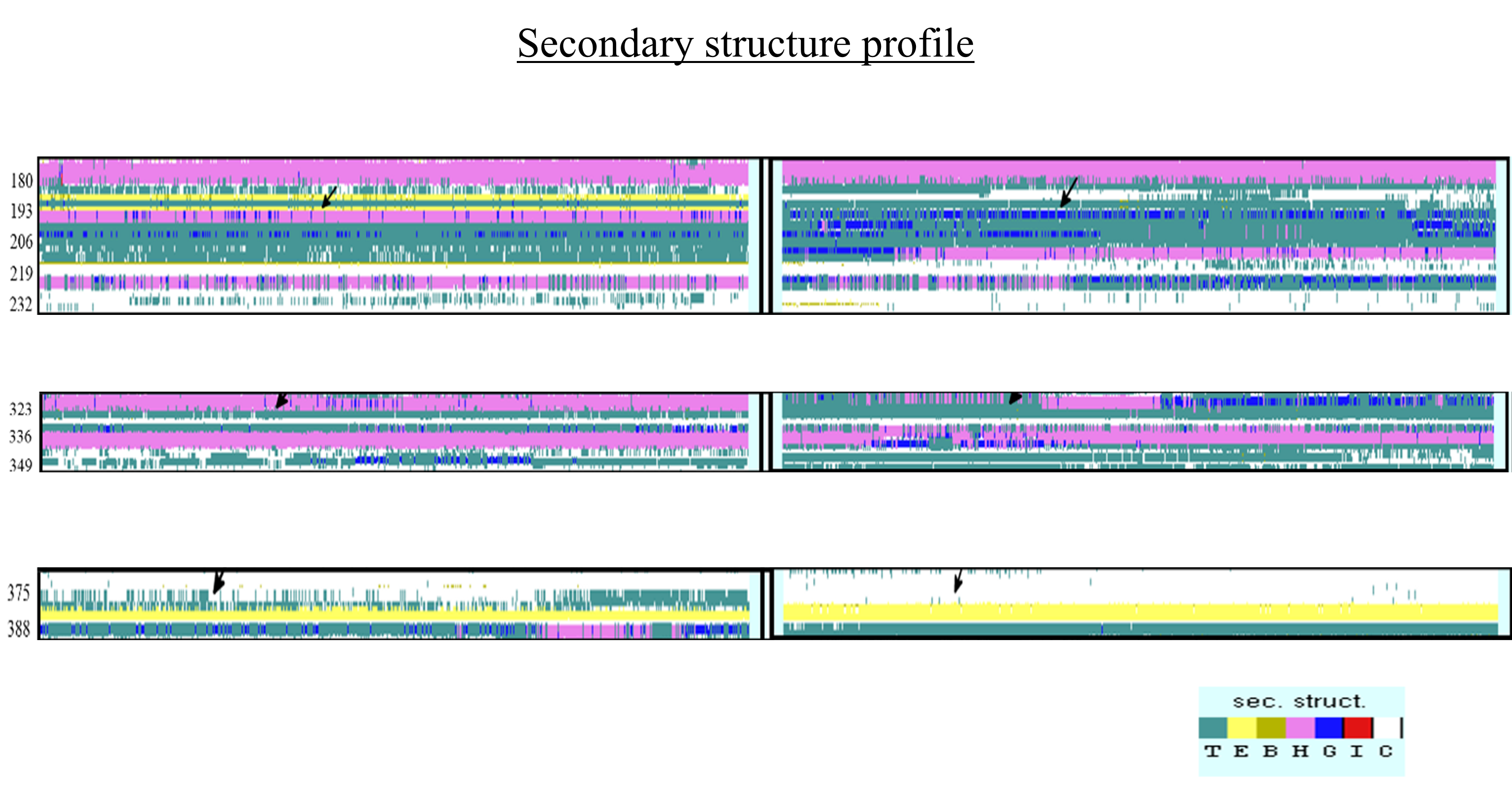
***Supplementary Fig. 6:*** The figure highlights the three major differences in secondary structural pattern that was observed during the MD run.


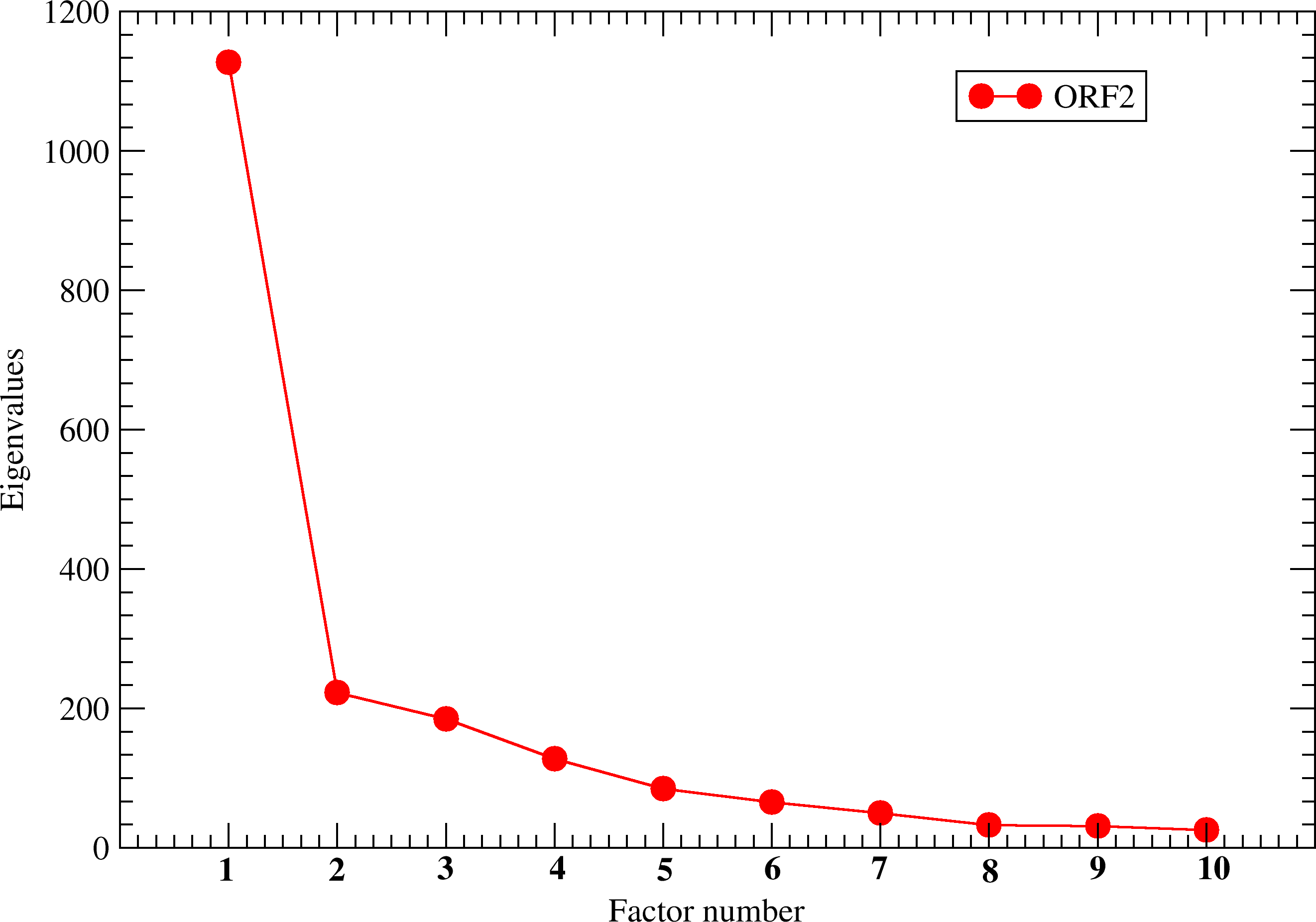

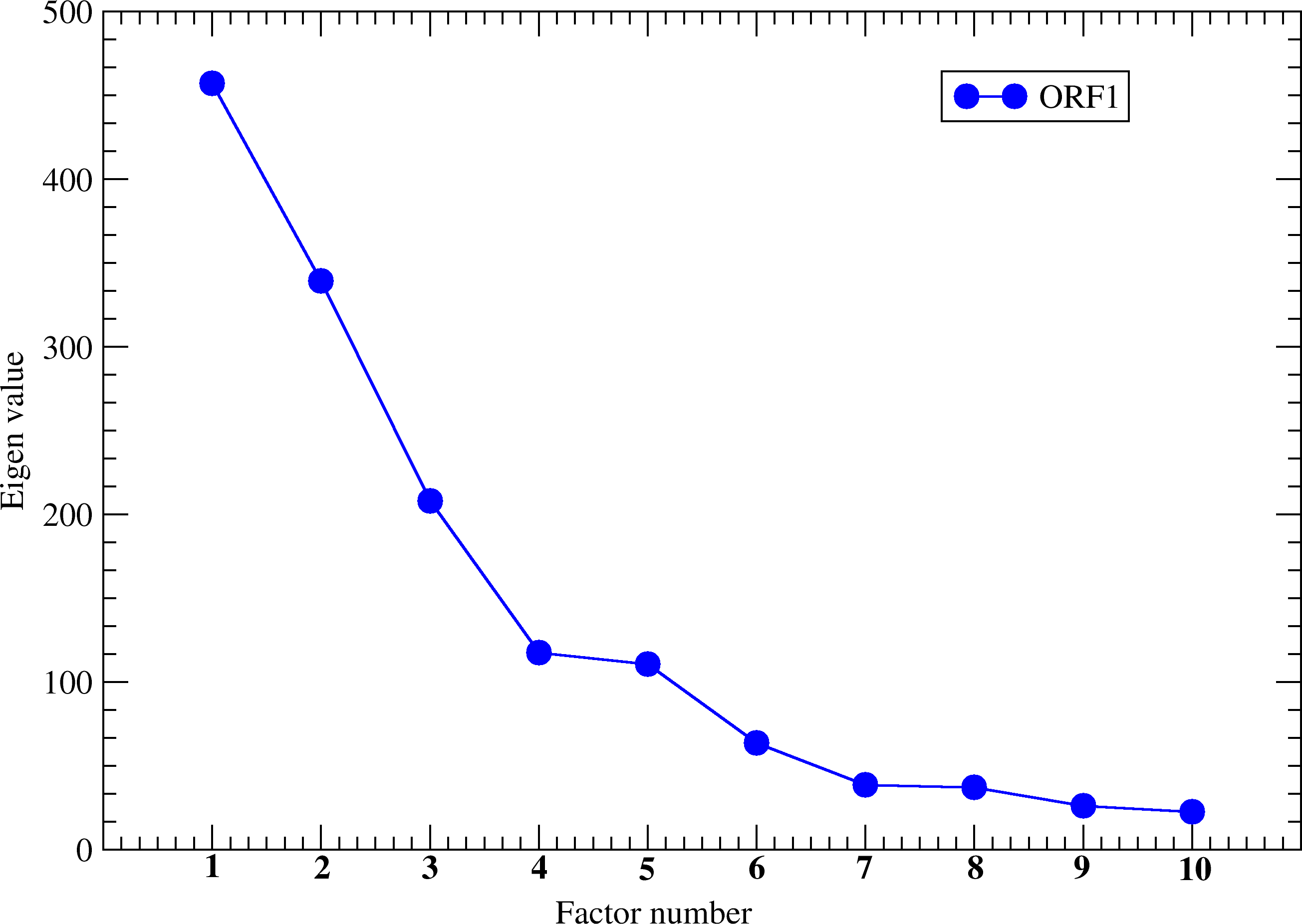


***Supplementary Fig. 7A:*** The figure represents the scree plot for the principal component analysis of both the ORFs.


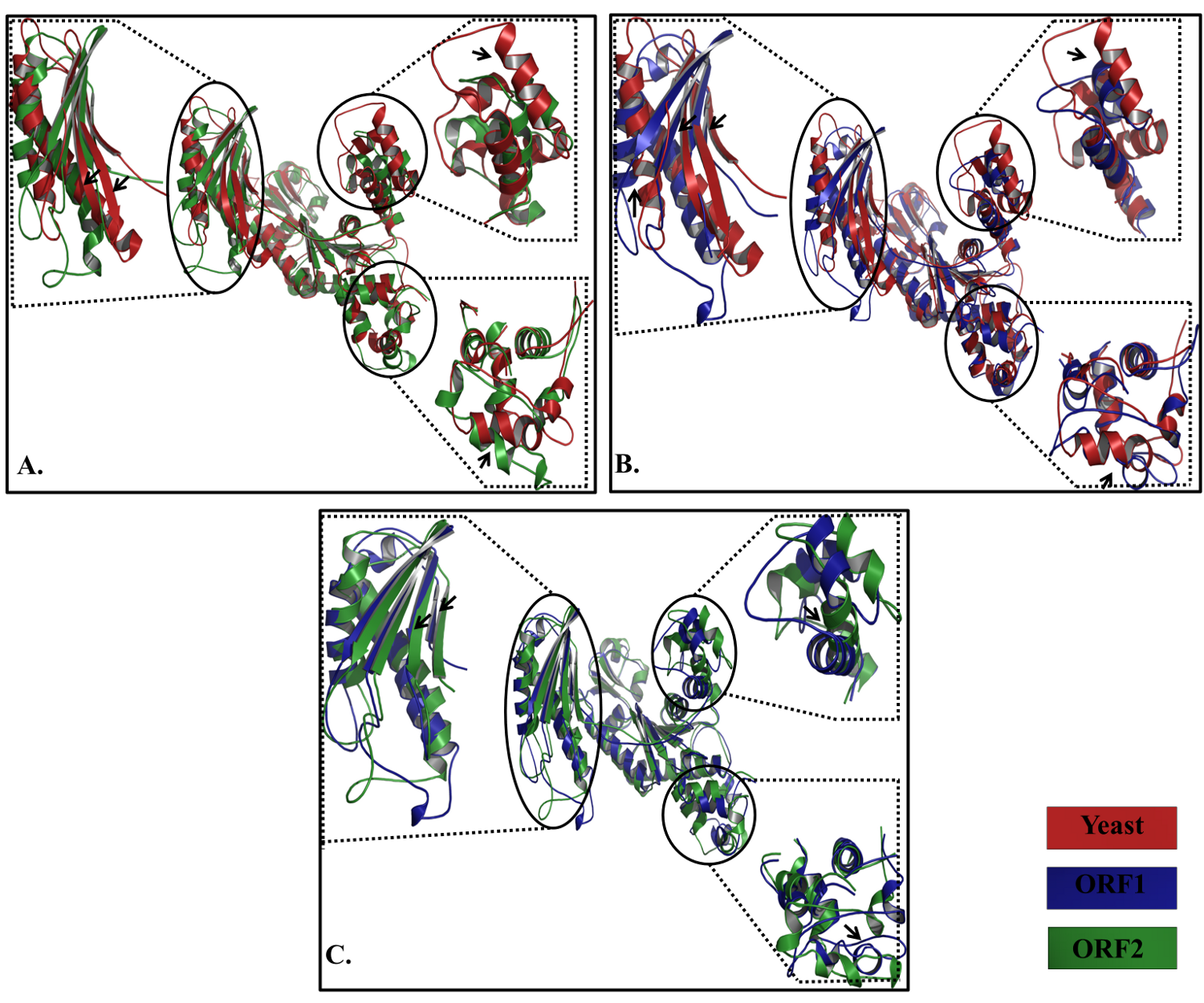
***Supplementary Fig. 7B:*** The figure represents the mean structural difference during the MD. The average structures (ORF1 and ORF2) from the largest cluster were superimposed to identify structural differences. The reference (PDB: 2WTF) structure is shown in red colour; ORF1 in blue colour and ORF2 in Green colour. **[**A] The average 3D structure of ORF2 superimposed on reference, [B] ORF1 superimposed on reference and [C] ORF1 superimposed on ORF2. The closer view (dashed boxes) of each domain is shown and variations were arrow marked.


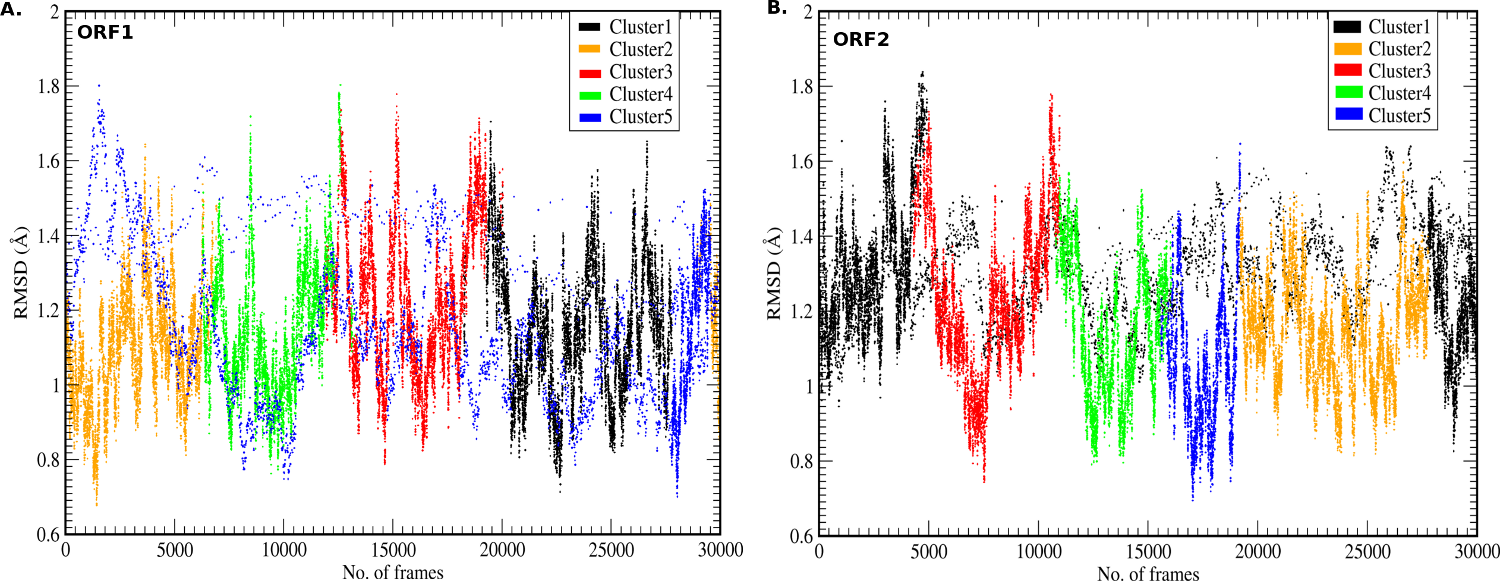
***Supplementary Fig. 8:*** The plot represents the five major clusters corresponds to the RMSD values for each frame throughout the trajectory. Each cluster is shown in different colour for both the ORFs.


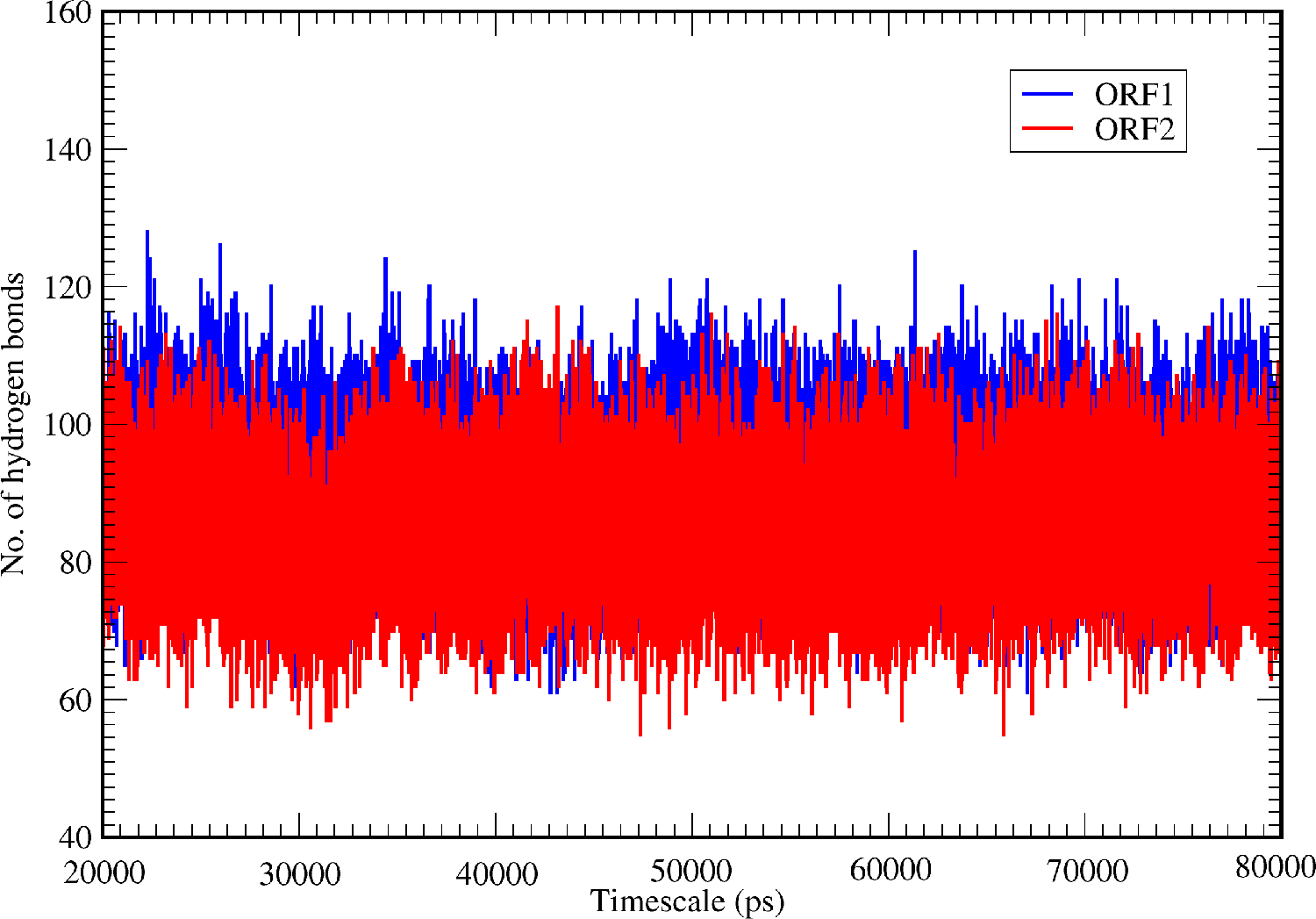
***Supplementary Fig. 9:*** The figure depicts the calculated total hydrogen bonds for the two ORFs throughout the simulation. The time step (ps) is shown on ‘X’- axis while number of hydrogen bonds is shown on ‘Y’- axis.


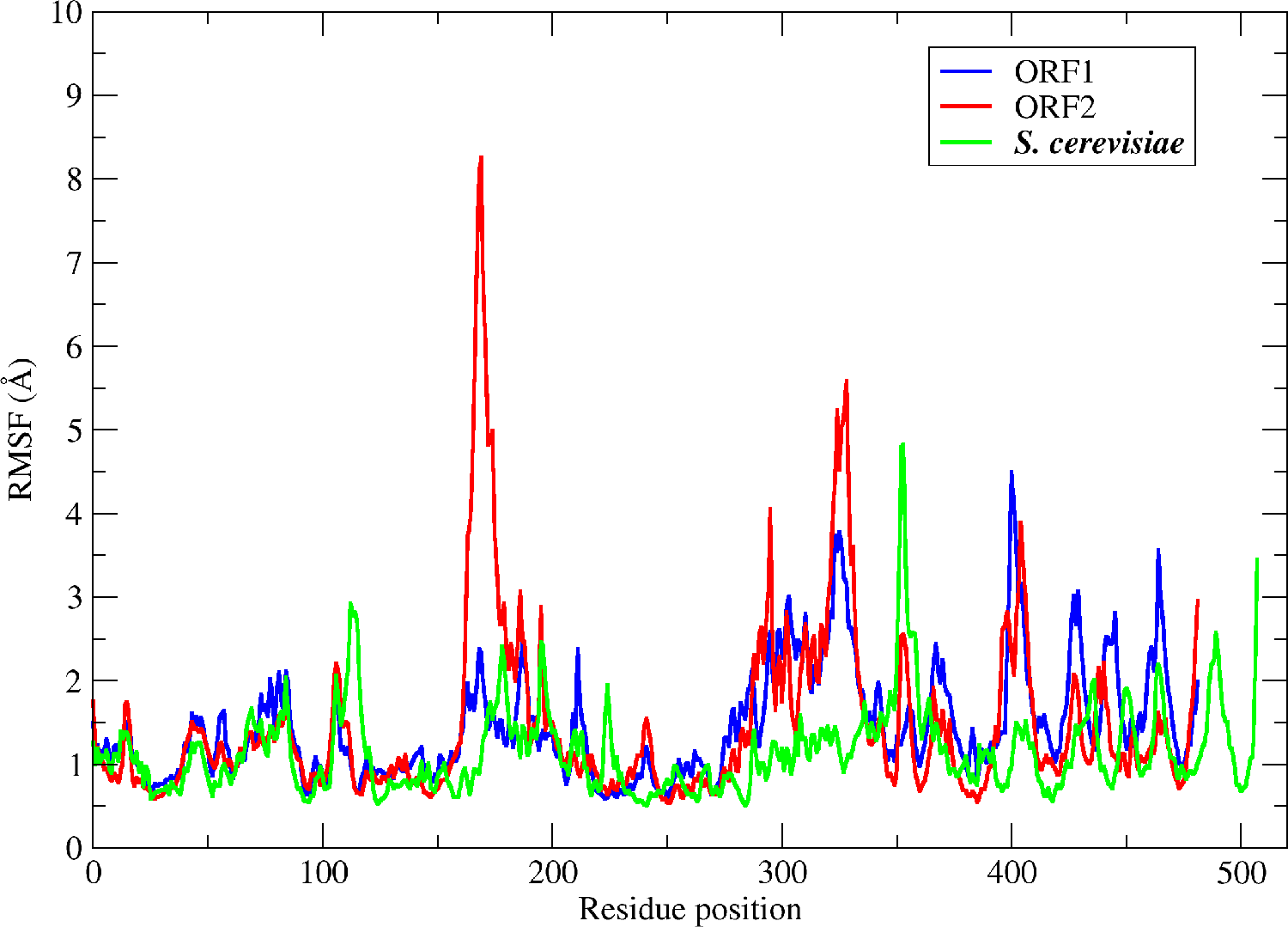
***Supplementary Fig. 10:*** The RMSF plot represents the comparison of RMSF values for the two ORFs with *S. cerevisiae* Polƞ system. The residue position and the RMSF (Å) are shown on ‘X’ and ‘Y’ - axis respectively.


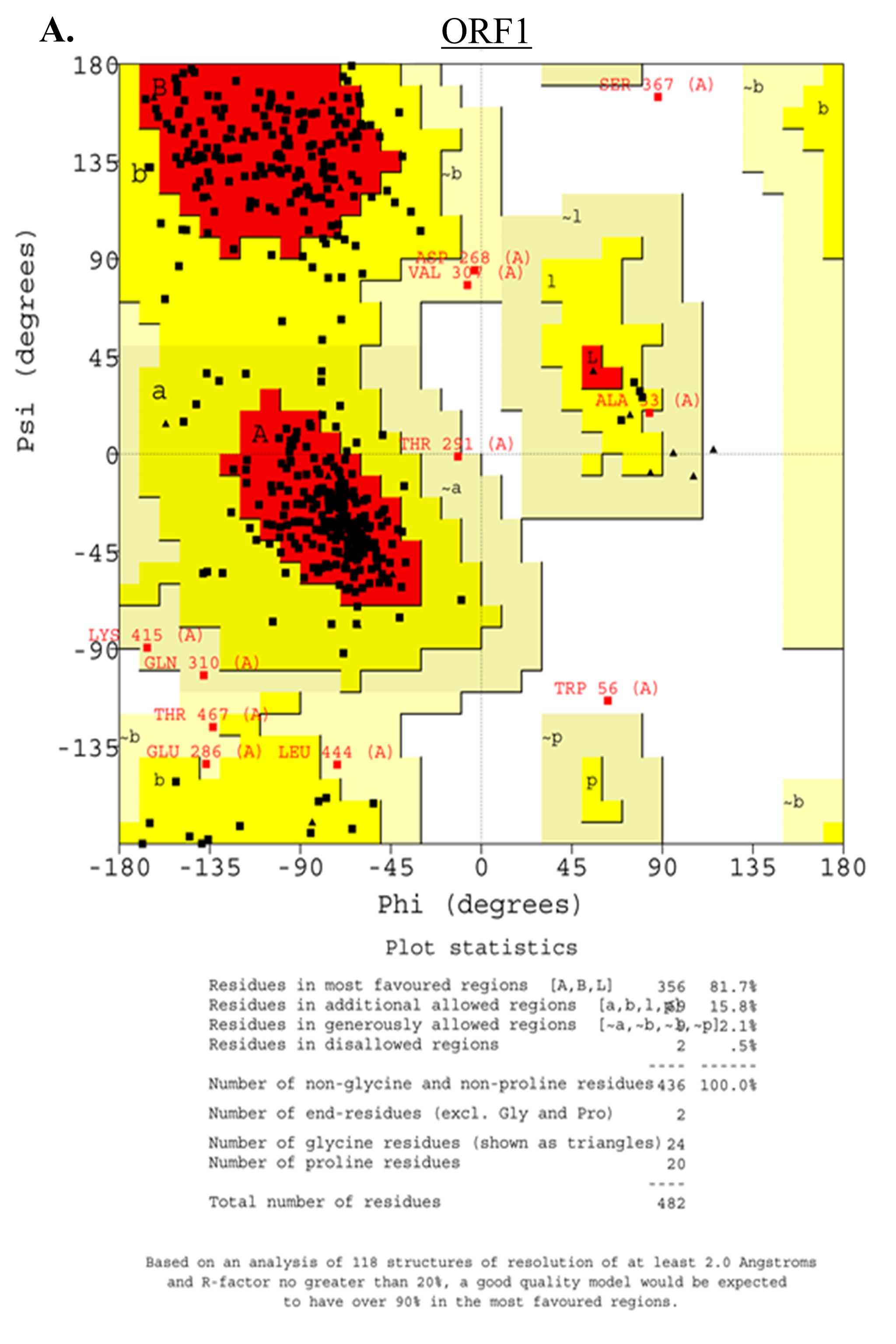

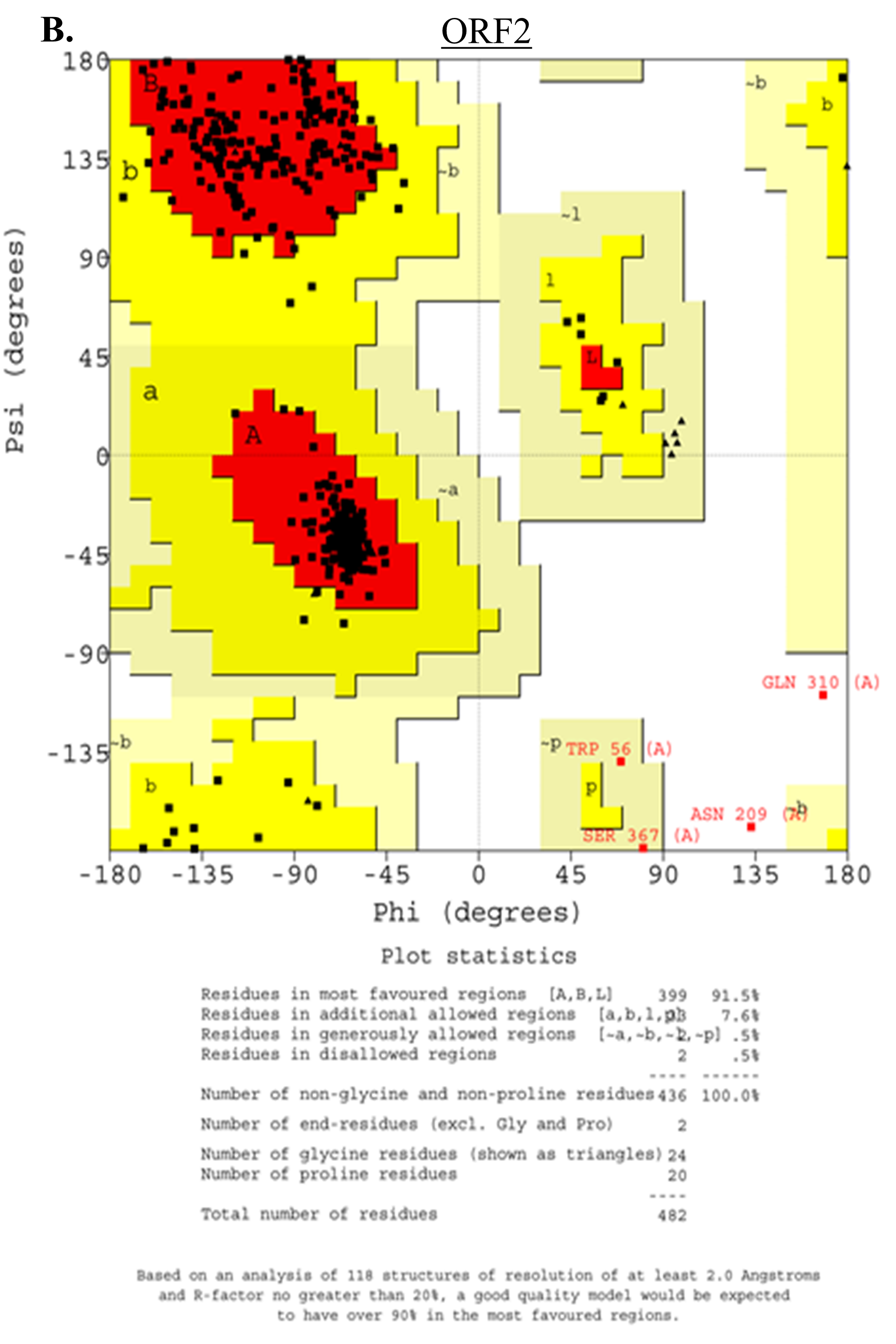
***Supplementary Fig. 11:*** The plot A & B represents the protein structure validation for the two ORFs using Ramachandran plot. The ‘X’ axis indicates Phi angle while ‘Y’ axis indicates Psi angle. The red zone indicates favored region while yellow indicates allowed region.

**Supplementary Table**

***Supplementary Table S1.* The distribution of conserved amino acids in different motifs.**

| **S. No.** | **Motif** | **Conservedper total residues** | **Location** | **Amino acid distribution** | | | | |
| --- | --- | --- | --- | --- | --- | --- | --- | --- |
|  |  |  |  | **Positively**  **Charged** | **Negatively**  **Charged** | **Non-polar** | **Aromatic** | **Polar** |
| 1. | **Motif-I** | 5/16 | β_1_→α_C_ (Palm) | - | D53, E62 | V61 | F57 | Q60 |
| 2. | **Motif-II** | 7/30 | β_3_→α_D_→ α_E_ (Finger) | R90, R96 | - | A84, V85, G93, C107 | Y87 | - |
| 3. | **Motif-III** | 6/14 | β_7_ (Palm) | - | E163, D168, E169, D173 | A165 | - | S166 |
| 4. | **Motif-IV** | 10/38 | α_J_→ β_10_→α_K_→ β_11_ (Palm) | R259,K278, K282, K289, | - | G273, L283, P290 | - | S271, Q293, T294 |
| 5. | **Motif-V** | 3/14 | α_N_→ α_M_ (Thumb) | - | - | G317, G318, G321 | - | - |
|  | **Total Residues** | | **31** | **6** | **6** | **12** | **2** | **5** |

***Supplementary Table S2.*** The occupancy of various π- π interactions in the two ORFs during dynamics.

| Residues | ORF1 | ORF2 | Location |
| --- | --- | --- | --- |
| H124_W125 | 100 | 100 |  |
| F57_Y268 | 100 | 100 | F57 (Motif-I) |
| F304_F308 | 100 | 100 |  |
| W402_F388 | 100 | 100 | F388( Patch-X) |
| F304_F287 | 100 | 100 | F287 (Motif-IV) |
| W125_Y118 | 100 | 100 |  |
| F58_Y144 | 100 | 100 | F58 (Motif-I) - Y144( Patch-V) |
| F308_F287 | 100 | 100 | F287 (Motif-IV) |
| Y118_H124 | 100 | 100 |  |
| Y144_Y127 | 100 | 100 | Y144( Patch-V) |
| F406_F388 | 100 | 100 | F388( Patch-X) |
| F475_Y400 | 100 | 100 |  |
| Y201_F186 | 0 | 99.47 |  |
| F29_W243 | 0 | 100 |  |
| Y144_Y127 | 100 | 100 | Y144( Patch-V) |
